# Viral Community Structure and Potential Functions in the Dried-Out Aral Sea Basin Change along a Desiccation Gradient

**DOI:** 10.1128/msystems.00994-22

**Published:** 2023-01-10

**Authors:** Wisnu Adi Wicaksono, Dilfuza Egamberdieva, Tomislav Cernava, Gabriele Berg

**Affiliations:** a Institute of Environmental Biotechnology, Graz University of Technology, Graz, Austria; b Institute of Fundamental and Applied Research, National Research University (TIIAME), Tashkent, Uzbekistan; c Leibniz Institute for Agricultural Engineering and Bioeconomy (ATB), Potsdam, Germany; d Institute for Biochemistry and Biology, University of Potsdam, Potsdam, Germany; University of California—San Diego

**Keywords:** viral communities, metagenomics, extreme environments, pioneer plants, *Suaeda acuminata*

## Abstract

The dried-out Aral Sea basin represents an extreme environment due to a man-made ecological disaster. Studies conducted in this unique environment revealed high levels of pollution and a specifically adapted microbiota; however, viral populations remained entirely unexplored. By employing an in-depth analysis based on the sequencing of metagenomic DNA recovered from rhizosphere samples of Suaeda acuminata (C. A. Mey.) Moq. along a desiccation gradient of 5, 10, and 40 years, we detected a diverse viral community comprising 674 viral populations (viral operational taxonomic units [vOTUs]) dominated by *Caudovirales*. Targeted analyses highlighted that viral populations in this habitat are subjected to certain dynamics that are driven mainly by the gradient of desiccation, the corresponding salinity, and the rhizosphere bacterial populations. *In silico* predictions linked the viruses to dominant prokaryotic taxa in the Aral Sea basin, such as *Gammaproteobacteria*, *Actinomycetia*, and *Bacilli*. The lysogenic lifestyle was predicted to be predominant in areas that dried out 5 years ago, representing the early revegetation phase. Metabolic prediction of viral auxiliary metabolic genes (AMGs) suggests that viruses may play a role in the biogeochemical cycles, stress resilience, and competitiveness of their hosts due to the presence of genes that are involved in biofilm formation. Overall, our study provides important insights into viral ecology in an extreme environment and expands our knowledge related to virus occurrence in terrestrial systems.

**IMPORTANCE** Environmental viruses have added a wealth of knowledge to ecological studies with the emergence of metagenomic technology and approaches. They are also becoming recognized as important genetic repositories that underpin the functioning of terrestrial ecosystems but have remain moslty unexplored. Using shotgun metagenome sequencing and bioinformatic tools, we found that the viral community structure was affected during natural revegetation in the dried-up Aral Sea area, a model habitat for investigating natural ecological restoration but still understudied. In this study, we highlight the importance of viruses, elements that are overlooked, for their potential contribution to terrestrial ecosystems, i.e., nutrient cycles, stress resilience, and host competitiveness, during natural revegetation.

## INTRODUCTION

The desertification of the Aral Sea basin in Uzbekistan and Kazakhstan is considered one of the most catastrophic environmental disasters of the last century (1). At present, the man-made terrestrial desert is characterized by distinct extremophilic conditions as well as the accumulation of various hazardous substances and heavy metals, i.e., Pb, Ni, Cu, and Cd (1–4). Over the past 40 years, the dried-out Aral Sea basin has undergone major changes, including a primary succession of halophilic plants (5). Therefore, this habitat provides an interesting model ecosystem for studying natural ecological restoration. Various studies have already addressed ecological changes in the Aral Sea basin, i.e., salinity levels, temperature fluctuations, and soil physicochemical properties (3, 6, 7). The first analyses of the water and soil of the still shrinking Aral Sea revealed microbial communities related to hypersaline-adapted bacteria and archaea; most of the archaeal sequences were phylogenetically affiliated with the order *Halobacteriales* but also indicated the presence of novel lineages (3, 8). Recent findings showed the importance of the members of the local plant-associated microbiota, especially those inhabiting the below-ground compartments, for ecosystem functioning during natural restoration (9, 10). Microbes can accelerate the decomposition of litter and the circulation of soil nutrients; both processes are important for soil multifunctionality during restoration. However, the complex interplay among microorganisms and interactions within their habitat can contribute substantially to ecosystem functioning, and therefore, the entire microbiome should be analyzed in ecosystem studies (11).

Recent bioinformatic developments allow us to analyze complex data sets from various habitats that include viral fragments and are therefore highly suitable for exploring factors that influence viral community dynamics (12, 13). Since the advent of metagenomic technologies and methodologies, environmental viruses have added a plethora of knowledge to ecological research. Viral communities have been intensively investigated in aquatic systems but are still poorly understood in terrestrial ecosystems (14). Marine viruses lyse over one-third of all ocean microorganisms every day, releasing substantial amounts of carbon and nutrients on a global scale (15–18). Numerous studies also indicated that viruses can carry auxiliary metabolic genes (AMGs), which are likely to play important roles in their prokaryotic host’s metabolism (19–21). Recent studies also demonstrated the potential roles of phages in the redistribution of plant-derived carbon into the rhizosphere environment through bacterial cell lysis (22). Accordingly, we expected analogous contributions to plant growth and nutrient cycling in the dried-out Aral Sea basin and that viruses would provide new clues to understanding ecosystem functioning and natural revegetation under extreme conditions.

This study centers on prokaryotic viral abundance and diversity in the dried-out Aral Sea basin. We investigated the structural and functional characteristics of viral communities that are associated with a common halophyte, Suaeda acuminata (C. A. Mey.) Moq. We selected S. acuminata (C. A. Mey.) Moq. because it is the first indigenous pioneer plant to naturally colonize various extreme environments (23), including the Aral Sea basin. By implementing shotgun metagenome sequencing and bioinformatic tools, we attempted to address the following questions: (i) Does a gradient of desiccation and the corresponding salinity shape viral community structures? (ii) Will we find a correlation between the viral community and the prokaryotic microbiota in the rhizosphere? (iii) Do viruses potentially provide a genetic reservoir of beneficial functions for their hosts in this harsh environment? To answer these questions, we obtained rhizosphere samples of the common halophyte and indigenous pioneer plant *S. acuminata* (C. A. Mey.) Moq. from areas that dried out 5, 10, and 40 years ago near the Large Aral Sea’s west shoreline to study interactions between the host plant and its associated microorganisms, including prokaryotic viruses. These areas were characterized by gradients of salinity and plant diversity (3). Moreover, microbial communities followed that gradient; dominant *Archaea* were replaced by *Bacteria* in the older parts of the basin (24). Here, we provide new evidence that viral communities in the dried-out Aral Sea basin are highly diverse and affected by a gradient of desiccation. The viral communities are potentially providing genetic reservoirs of beneficial functions for their hosts to survive in this harsh environment.

## RESULTS

### Temporal dynamics of the viral community along the desiccation gradient.

**FIG 1:**
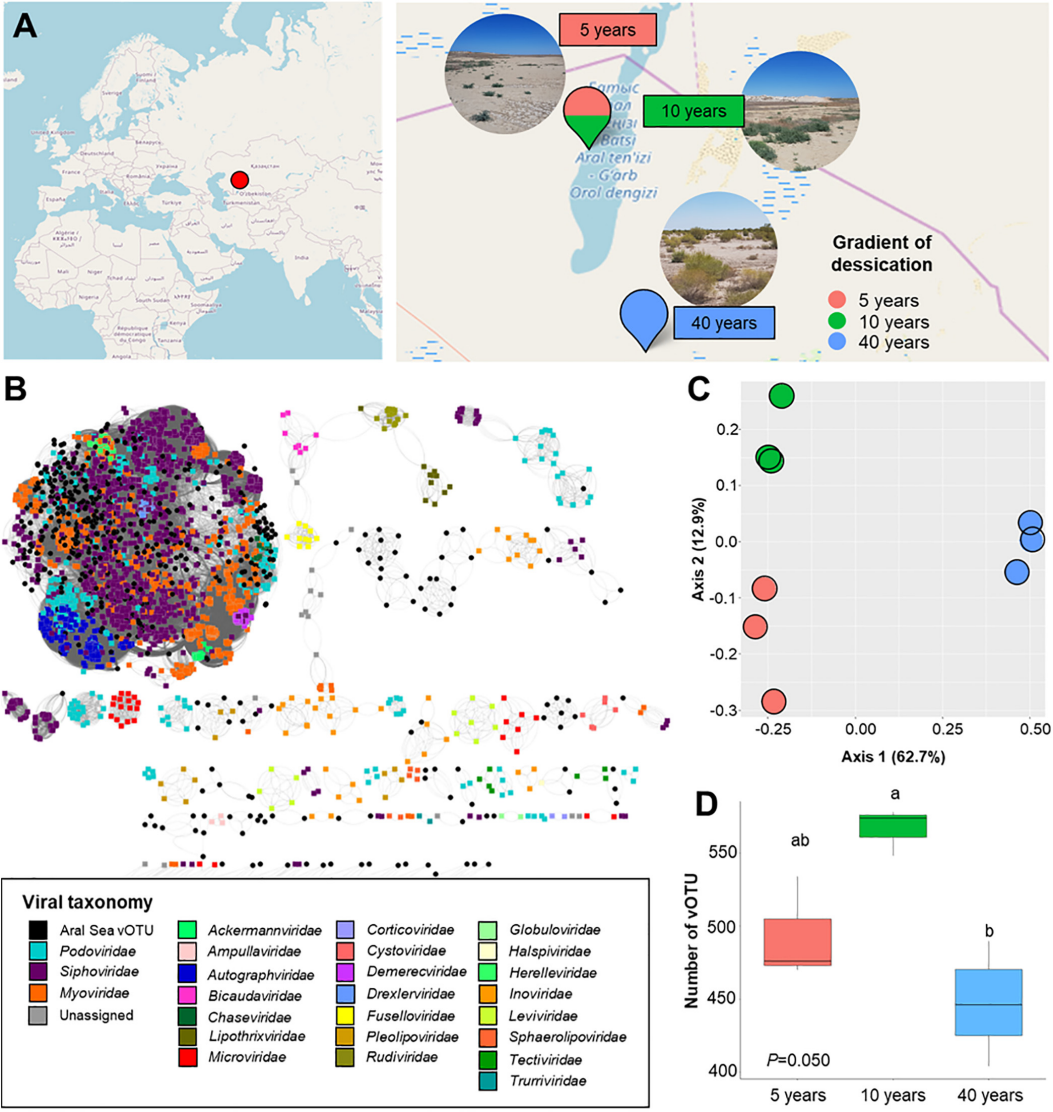
Viral taxonomic information, diversity, and community structure of the rhizosphere of *Suaeda acuminata* along the desiccation gradient. (A) Sampling points within the dried-out Aral Sea basin. GPS data were visualized using OpenStreetMap. (B) Gene-sharing network for vOTUs of >10 kb from the dried-out Aral Sea basin (black circles) and RefSeq prokaryotic viral genomes (colored circles). (C) Principal-coordinate analysis (PCoA) showing clustering of the viral community structures (based on vOTUs), based on a Bray-Curtis distance matrix. (D) Differences in alpha diversity (number of vOTUs detected) within the analyzed desiccation gradient. Significances in the numbers of detected vOTUs in panel C, as indicated by different letters, representing *P* values of <0.05, were determined by a pairwise Wilcox test.

We analyzed nine metagenomes from three different sampling sites representing a gradient of desiccation and revegetation in the dried-out basin of the South Aral Sea (Fig. 1A). After *de novo* assembly and the removal of redundant contigs, 674 viral operational taxonomic units (vOTUs) were identified, with a minimum length of 10 kb and a maximum length of 312 kb. According to CheckV, totals of 11 and 10 vOTUs were estimated to be complete and high-quality viral genomes, respectively (see Table S1 in the supplemental material). vConTACT2 was used to cluster the Aral Sea vOTUs and sequences from the prokaryotic ViralRefSeq 201 database. This analysis yielded 104 viral clusters (VCs), where VCs approximate genus-level taxonomy, and 10 singletons. Only 12.3% (*n* = 14) of the VCs could be taxonomically assigned, which indicates the potential occurrence of as-yet-unknown viral taxa in populations of the dried-out Aral Sea basin. The majority of the known viral clusters were closely related to three viral families within the *Caudovirales* order. They included *Siphoviridae*, *Myoviridae*, and *Podoviridae.* The family *Pleolipoviridae* was identified with the order *Haloruvirales* (Fig. 1B).

10.1128/msystems.00994-22.3 TABLE S1 Details of the viral population (vOTUs). Download Table S1, DOCX file, 0.1 MB. Copyright © 2023 Wicaksono et al. 2023 Wicaksono et al. https://creativecommons.org/licenses/by/4.0/ This content is distributed under the terms of the Creative Commons Attribution 4.0 International license.

Based on the abundance of vOTUs, we observed that the desiccation gradient highly affected the viral community structure (*R*^2^ = 74.3% and *P* = 0.004 [by permutational multivariate analysis of variance {PERMANOVA}]). In a principal-coordinate analysis (PCoA) plot based on a Bray-Curtis distance matrix, the first two principal coordinates (PCs) explained 75.6% of the cumulative variance, which highlighted the significant differences between sample groups based on the desiccation gradient. Samples that were obtained from areas that dried out 5 and 10 years ago (early revegetation phase) tended to cluster closer than samples that were obtained in the area that dried out 40 years ago (late revegetation phase) (Fig. 1C). On the other hand, samples that were obtained from the area that dried out 40 years ago tended to cluster separately. Additionally, the community structure based on a Jaccard distance matrix indicated a pattern similar to the one mentioned above (*R*^2^ = 60.8% and *P* = 0.004 [by PERMANOVA]) (Fig. S1). The total viral abundances (summed reads per kilobase per million [RPKM] values for all viruses present in that sample) in the rhizosphere were relatively stable along the desiccation gradient (*P* = 0.252 [by a Kruskal-Wallis test]). Interestingly, the area that dried out 10 years ago harbored the highest number of vOTUs. We also observed a decrease in viral richness in the area that dried out 40 years ago in comparison to the area that dried out 10 years ago (*P* = 0.027 [by a Kruskal-Wallis test]) (Fig. 1D). Overall, we observed that the desiccation gradient was a significant factor that shaped the viral community structures.

10.1128/msystems.00994-22.1 FIG S1 Principal-coordinate analysis (PCoA) showing clustering of the viral populations (based on vOTUs), based on a Jaccard distance matrix. Download FIG S1, TIF file, 0.1 MB. Copyright © 2023 Wicaksono et al. 2023 Wicaksono et al. https://creativecommons.org/licenses/by/4.0/ This content is distributed under the terms of the Creative Commons Attribution 4.0 International license.

### The viral community structures were associated with specific bacterial host lineages.

We further explored the potential associations between the viral and prokaryotic community profiles. Using amplicon sequencing data that were generated from the same samples, we observed a high correlation (*r* = 0.885 and *P* = 0.001 [by a Mantel test]) between the prokaryotic community structure and the viral community structure. However, we did not find a significant correlation between the number of vOTUs and the number of prokaryotic species (Pearson *R* = −0.280 and *P* = 0.460) and diversity estimated using the Shannon index (Pearson *R* = −0.520 and *P* = 0.160). From the metagenomic data, we recovered a total of 112 medium- to high-quality bacterial genomes with high completeness (≥75%) and contaminations of <10% from the shotgun-sequenced data set (Table S2). The majority of these metagenome-assembled genomes (MAGs) were assigned to *Gammaproteobacteria* (*n* = 35), *Actinomycetia* (*n* = 19), *Halobacteria* (*n* = 13), *Alphaproteobacteria* (*n* = 12), *Bacteroidia* (*n* = 7), *Rhodothermia* (*n* = 5), and *Gemmatimonadetes* (*n* = 5).

10.1128/msystems.00994-22.4 TABLE S2 Details of metagenome-assembled genomes (MAGs). Download Table S2, DOCX file, 0.03 MB. Copyright © 2023 Wicaksono et al. 2023 Wicaksono et al. https://creativecommons.org/licenses/by/4.0/ This content is distributed under the terms of the Creative Commons Attribution 4.0 International license.

**FIG 2:**
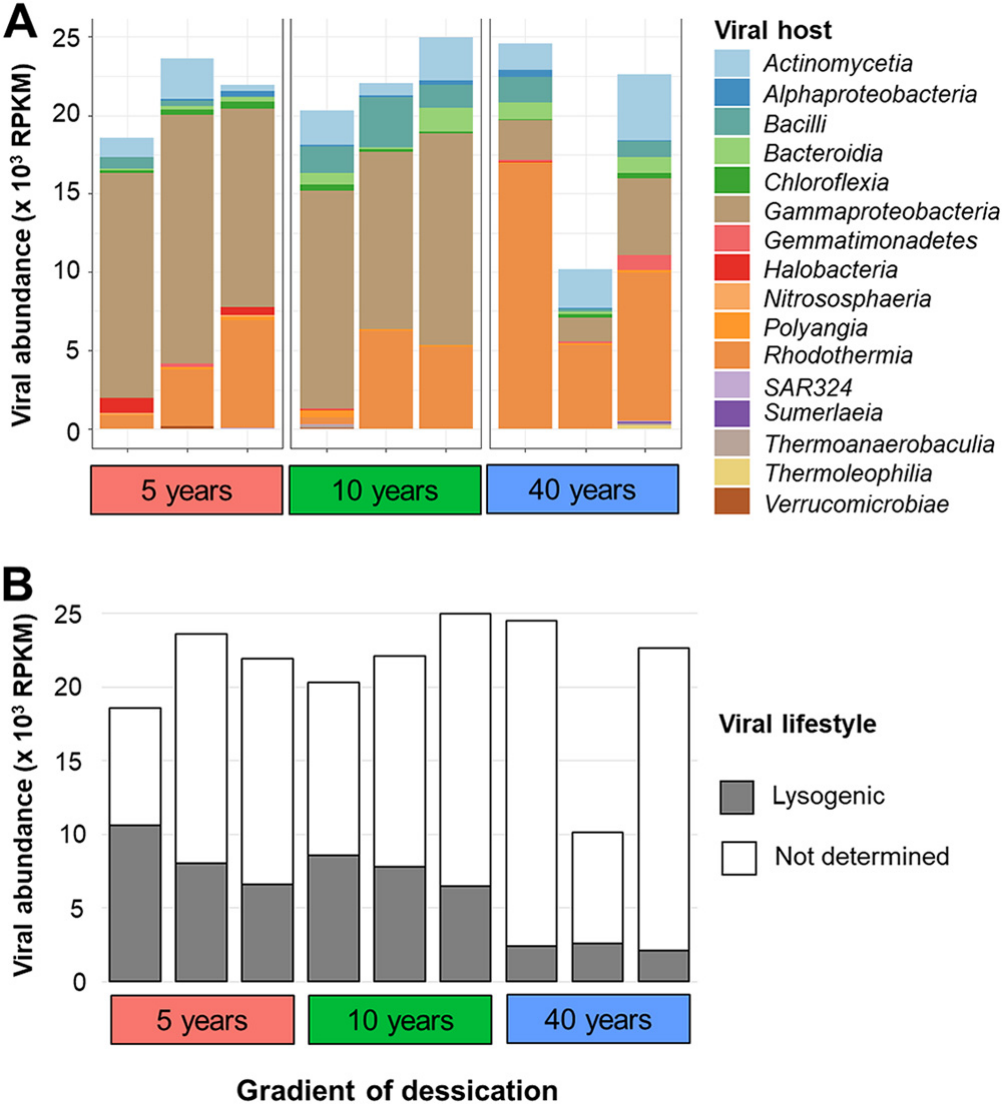
Abundance profiling of vOTUs along a desiccation gradient. The bar plot shows the abundances (RPKM) of vOTUs that were grouped according to viral host (A) and viral lifestyle (B).

Using the abundance profiles of MAGs, we confirmed the high correlation between the viral community structure and the microbial community structure (*r* = 0.911 and *P* = 0.001 [by a Mantel test]). Next, we attempted to predict the potential microbial hosts of the detected viruses using three different *in silico* approaches based on CRISPR spacers, oligonucleotide frequencies (ONFs), and sequence similarity. In total, we recovered 831 CRISPR spacers in 112 MAGs and identified 13 vOTUs that were linked to 12 MAGs that belong to *Gammaproteobacteria* (*n* = 5), *Actinomycetia* (*n* = 2), *Alphaproteobacteria* (*n* = 2), *Bradymonadia* (*n* = 2), *Bacilli* (*n* = 1), and *Halobacteria* (*n* = 1) (Table S1). We furthermore found that 46 vOTUs were linked to 26 MAGs, where the majority were assigned to *Gammaproteobacteria* (*n* = 12), *Bacilli* (*n* = 9), *Actinomycetia* (*n* = 8), and *Alphaproteobacteria* (*n* = 8) based on nucleotide sequence homology. The majority of the potential microbial hosts of the detected viruses was identifiable only using VirHostMatcher (25). We identified putative interactions ($d_{2}^{*}$ values of ≤0.17) between 615 vOTUs and their putative prokaryotic hosts. High proportions of vOTUs were linked to *Gammaproteobacteria* (*n* = 288), followed by *Actinomycetia* (*n* = 104) and *Rhodothermia* (*n* = 52) (Table S1). The abundance of *Halobacteria* showed a tendency to decrease along the gradient of desiccation. A similar pattern was observed for the relative abundances of viruses that were predicted to infect the above-mentioned prokaryotic hosts (Fig. 2A). Interestingly, *Gammaproteobacteria* were highly abundant in the rhizosphere samples of the area that dried out 5 years ago and gradually decreased along the gradient of desiccation, whereas *Actinomycetia* showed the opposite pattern (Fig. S2). Correlation analysis indicated that the abundances of *Gammaproteobacteria* and *Halobacteria* were correlated with the abundances of viruses that were predicted to infect them (*Gammaproteobacteria*, Pearson *R* = −0.820 and *P* = 0.007; *Halobacteria*, Pearson *R* = 0.840 and *P* = 0.005). A weak correlation was observed between the abundance of *Actinomycetia* and the abundance of viruses that were predicted to infect them (Pearson *R* = −0.590 and *P* = 0.095). This observation indicated that the viral community structure was driven by specific bacterial host lineages. Based on the lifestyle assessment of the viruses using BACPHLIP, only 25.8% of the vOTUs (*n* = 174) (Table S1) were predicted to have a lysogenic lifestyle. The abundance of lysogenic phages showed a tendency to decrease along the desiccation gradient (*P* = 0.060 [by a Kruskal-Wallis test]) (Fig. 2B).

10.1128/msystems.00994-22.2 FIG S2 Abundance profiling of metagenome-assembled genomes (MAGs) from a desiccation gradient in the Aral Sea basin. Download FIG S2, TIF file, 0.2 MB. Copyright © 2023 Wicaksono et al. 2023 Wicaksono et al. https://creativecommons.org/licenses/by/4.0/ This content is distributed under the terms of the Creative Commons Attribution 4.0 International license.

### Viral AMGs are potentially involved in carbon cycling, sulfur and cofactor/vitamin metabolism, and the biosynthesis of secondary metabolites.

**FIG 3:**
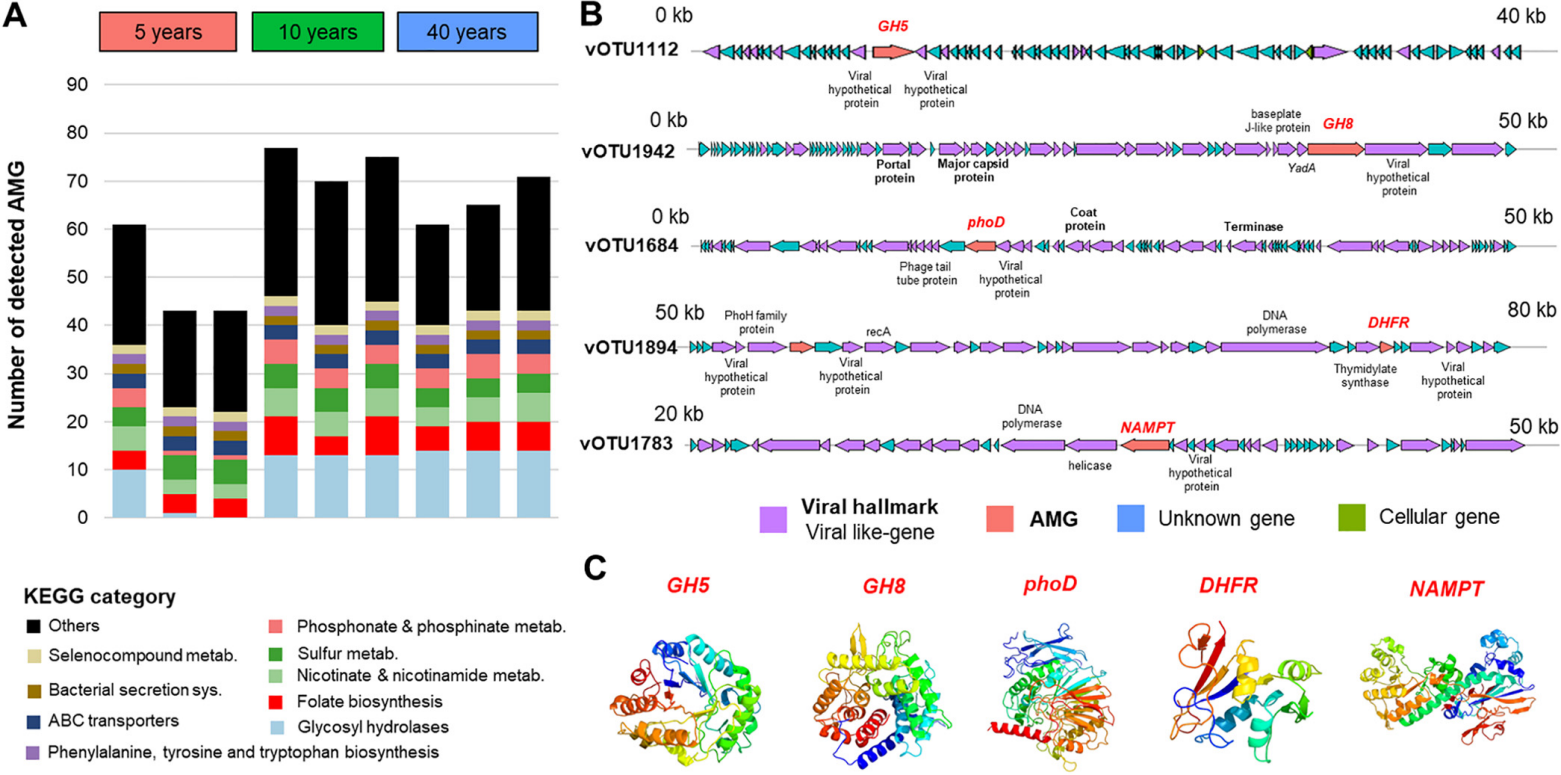
Detected virus auxiliary metabolic genes (AMGs). (A) Bar plot showing the number of AMGs that were present at each sampling location. (B and C) Visualization of the genomic context of representative AMG-carrying viruses (B) and predicted protein structures with the AMGs of interest based on structural modeling using Phyre2 (C).

To infer the potential ecological importance of the viruses, we further examined viral auxiliary metabolic genes (AMGs) that may support host metabolism during infection. According to DRAM-v and VIBRANT, we detected AMGs from 62 vOTUs. Many of the viruses containing the AMGs were predicted to infect *Gammaproteobacteria* (*n* = 23) and *Actinomycetia* (*n* = 14) (Table S3 and Data Set S1). Based on *in silico* analyses of viral proteins, all selected AMGs contained conserved functional domains and the structural configuration (confidence of >97%) (Table S4) of enzymes that are involved in carbon, phosphate, cofactor, and vitamin metabolism (Fig. 3A). Genes involved in carbon cycling were detected in 23 vOTUs. We also detected genes encoding enzymes that catalyze the initial breakdown of complex polysaccharides such as cellulose and chitosan, i.e., *GH5*, *GH6*, *GH8*, and *PL7* (Fig. 3B and C, Data Set S1, and Table S4). The majority of these vOTUs were predicted to infect *Actinomycetia*, *Gammaproteobacteria*, and *Bacilli* (Table S3). Interestingly, the number of genes that are involved in the initial breakdown of complex polysaccharides was higher in the areas that dried out 10 and 40 years ago than in the area that dried out 5 years ago (Fig. 3A). The presence of *phnP*, which encodes phosphoribosyl 1,2-cyclic phosphate phosphodiesterase (KO06167), in five vOTUs and *phoD*, which encodes alkaline phosphatase D (K01113), in two vOTUs indicated that these genes may contribute to phosphate solubilization by their host. Moreover, we also identified a gene involved in sulfur metabolism, *cysH*, which encodes phosphoadenosine phosphosulfate reductase (K00390) and was found in four vOTUs that were linked to *Gammaproteobacteria*, *Actinomycetia*, and *Halobacteria*. The presence of genes that encode dihydrofolate reductase (*DHFR*) (K00287) and nicotinamide phosphoribosyltransferase (*NAMPT*) (K03462) indicated the potential role of viruses in the metabolism of cofactors and vitamins such as tetrahydrofolate and nicotinamide. Overall, we found that viruses potentially mediate distinct biogeochemical processes in the dried-out Aral Sea basin.

10.1128/msystems.00994-22.5 TABLE S3 Details of auxiliary metabolic genes (AMGs) from the viral population (vOTUs). Download Table S3, DOCX file, 0.03 MB. Copyright © 2023 Wicaksono et al. 2023 Wicaksono et al. https://creativecommons.org/licenses/by/4.0/ This content is distributed under the terms of the Creative Commons Attribution 4.0 International license.

10.1128/msystems.00994-22.6 TABLE S4 Details about the manual curation of selected AMGs. Download Table S4, DOCX file, 0.02 MB. Copyright © 2023 Wicaksono et al. 2023 Wicaksono et al. https://creativecommons.org/licenses/by/4.0/ This content is distributed under the terms of the Creative Commons Attribution 4.0 International license.

10.1128/msystems.00994-22.8 DATA SET S1 Details about the genomic context of representative AMG-carrying viruses. Download Data Set S1, XLSX file, 0.2 MB. Copyright © 2023 Wicaksono et al. 2023 Wicaksono et al. https://creativecommons.org/licenses/by/4.0/ This content is distributed under the terms of the Creative Commons Attribution 4.0 International license.

AMGs that are likely involved in bacterial competitiveness and tolerance against environmental stress were also detected. Genes that are involved in biofilm formation (*rfbD* [K00067]) and the branched-chain amino acid transport system (*livH*, *livK*, and *livM*) were detected in viruses that infect *Gammaproteobacteria*. We also identified a gene that encodes *p*-hydroxybenzoate 3-monooxygenase in one vOTU, which is potentially involved in soil detoxification by *Actinomycetia* as its putative prokaryotic hosts.

## DISCUSSION

**FIG 4:**
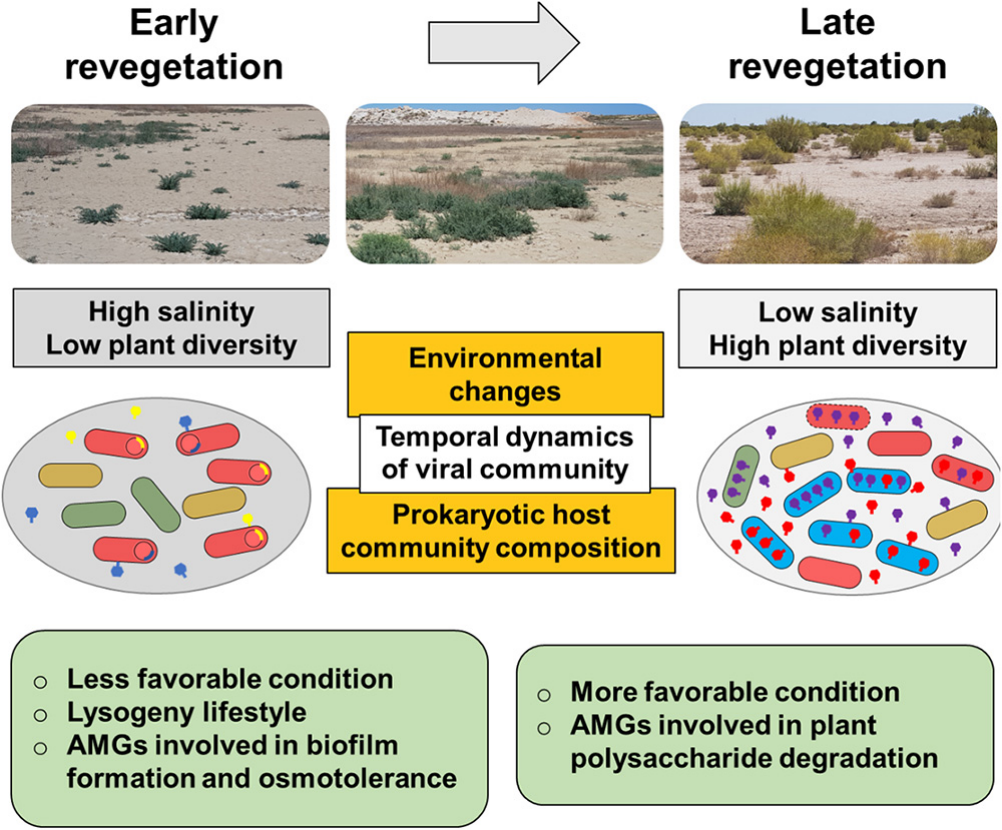
Hypothetical model of the dynamics of the viral community of the dried-out Aral Sea basin. Viruses with a lysogenic lifestyle were predominant in the areas representing the early revegetation phase. They were also found to potentially provide a genetic reservoir of beneficial functions for their hosts to survive under less favorable conditions.

Our in-depth assessment of prokaryotic viral diversity in the dried-out Aral Sea basin, one of the world’s most extreme environments due to rapid desiccation, high salinity, and the accumulation of toxic compounds, revealed viral richness shaped by the microbial host’s community structure. In general, viral diversity in extreme environments is still largely unexplored (26, 27). In comparison to other hyperarid and saline environments, i.e., the Atacama Desert (28, 29) and the Peñahueca shallow saline lake (30), a relatively high number of vOTUs was detected in the Aral Sea basin. Viruses that were linked to distinct hosts contained auxiliary metabolic genes (AMGs) that may play roles in the biogeochemical cycles, competitiveness, and resilience against environmental stress of their putative hosts. Overall, based on the assessment of viral composition and function profiles, we propose a hypothetical model of the dynamic responses of the viral community in the dried-out Aral Sea basin to host variations and environmental factors along the desiccation-and-revegetation gradient (Fig. 4).

One of the main observations of the present study was that viral community dynamics were driven by the desiccation gradient and specific bacterial host lineages. The main underlying factor could be differences in the salinity levels of the studied soil samples; they decreased along the gradient of desiccation (3). Salinity is a major factor shaping microbial community structures in desert ecosystems (3, 31–33). In this study, we observed a high correlation between the prokaryotic and viral community structures. Moreover, the majority of the viral populations were predicted to infect *Gammaproteobacteria*, *Actinomycetia*, and *Bacilli*, the dominant prokaryotic taxa in the dried-out Aral Sea basin. Accordingly, temporal changes in the viral community structure are likely connected to changes in the abundances of their putative prokaryotic hosts along the desiccation gradient. Despite the comprehensive analysis that was conducted, this study has certain limitations, such as the low numbers of biological replicates and sampling sites and missing metadata, i.e., soil pH, water content, soil nutrient content, and organic matter content, that might have impacts on viral community structures. More comprehensive studies with more samples will be required in the future to assess which factors within the desiccation gradient influence viral community structures.

Viruses with a lysogenic lifestyle dominated the Aral Sea basin in the area that dried out 5 years ago. In general, deterministic factors and ecological mechanisms of bacteriophage lifestyles remain mostly unclear (14, 34). Environmental conditions, i.e., nutrients, pH, or temperature, are suggested to influence bacteriophage lifestyles (35). It was also suggested that the lysogenic lifestyle is predominant in harsh environments (36). In a recent study of the viral community in the Atacama Desert, Hwang and colleagues (29) suggested that under less favorable conditions, viruses likely undergo lysogeny cycles to seek protection in their host cells as a survival strategy. In the present study, the area that dried out 5 years ago was the soil zone that was nearest to the present shoreline of the Aral Sea and can be considered a hostile environment for most organisms due to its high salinity (3) compared to other sampling areas. Therefore, due to less favorable conditions, a predominance of viruses with a lysogenic lifestyle was most likely detected in the area that dried out 5 years ago (Fig. 4). Despite the first evidence that certain factors influence bacteriophage lifestyles in the Aral Sea basin, results from the prediction tools utilized must be interpreted with caution because some of the analyzed viral genomes are not complete (37). As a result, this can lead to an underestimation of viruses that are lysogenic because relevant lysogeny-associated proteins might be encoded within the missing genome segments. Moreover, there are other lifestyles, such as pseudolysogeny, chronically infecting, and budding, that were not investigated in this study.

Viruses with auxiliary metabolic genes (AMGs) potentially provide a genetic reservoir of beneficial functions for their hosts (35, 38). Beneficial genes such as genes encoding the branched-chain amino acid transport system were detected in a virus from the dried-out Aral Sea basin that infects *Gammaproteobacteria*, a dominant taxon in the area that dried out 5 years ago. This transport system is involved in salt stress maintenance, possibly via the exchange of solutes across the membrane, and therefore increases the host’s osmotolerance (39). Moreover, the presence of genes that are involved in biofilm formation, i.e., *rfbD* (40, 41), in the viruses may increase the chances of their host surviving in this hostile environment (Fig. 4). Viruses that harbor AMGs that are potentially involved in plant polysaccharide degradation were more frequently identified in the area that dried out 40 years. According to the analyses conducted, these viruses were inferred to infect *Actinomycetia*, the naturally dominant bacterial taxon in this area. Members of the *Actinomycetia* are known as primary decomposers of plant organic matter, i.e., lignocellulose, xylan, and pectin (42, 43). Viruses with AMGs, i.e., glycoside hydrolases and a polysaccharide lyase, that are involved in plant polysaccharide degradation might increase their host’s fitness and abundance under local conditions (Fig. 4). Until now, only a few studies have experimentally validated the functions of AMGs, e.g., *psbA*, *pebS*, and glycoside hydrolase, by using biochemical assays (44–46). We acknowledge that although bioinformatics tools such as DRAM-v (47) and VIBRANT (48) provide automated ways to identify candidate AMGs, the results obtained in this study regarding AMGs need to be further verified, especially in terms of confirming that the candidate AMGs are truly carried by viruses and involved in bacterial metabolic pathways.

Overall, our study highlights the importance of virus-host interactions that can have potential implications for modulating microbially driven processes, i.e., carbon cycling and microbial strategies, to survive in the dried-out Aral Sea basin. Future studies based on virus-enriched metagenomes in combination with the isolation and cultivation of the identified viruses could further clarify their lifestyle and their detailed functional implications in this highly specific ecosystem.

## MATERIALS AND METHODS

### Sample collection and shotgun metagenomic sequencing.

The South Aral Sea belongs to Uzbekistan (45°00′N, 60°00′E) and previously had a surface area of 60,000 km^2^ but is continuously shrinking. We collected rhizosphere samples of the plant *S. acuminata* (C. A. Mey.) Moq. in the dried-out basin and near the west shoreline of the South Aral Sea. Rhizosphere samples were obtained from three sampling sites (three biological replicates from each site) that represent a gradient of desiccation from areas that dried out 5, 10, and 40 years ago (Fig. 1A).

These sampling locations were studied previously, and metadata for geochemistry and mineralogy were obtained (3). The soil contained sand, clay, and silt at 37.9%, 53.2%, and 8.9%, respectively. Between the region that dried out 5 years ago and the area that dried out 40 years ago, the gradient of salinity (total soluble salt) is huge and differed from 67.1 g/L to 0.4 g/L (Fig. 1A). Moreover, a negative correlation between salinity and the variety of plant species was observed in the studied region (3). Therefore, the area that dried out 5 years ago represents an early revegetation phase due to low plant diversity, while the area that dried out 40 years ago represents a late revegetation phase (Fig. 1A).

Prior to total DNA extraction, plant roots with adhering rhizosphere soil were mixed with 20 mL sterile 0.85% NaCl and homogenized by vortexing for 3 min. An aliquot of the samples (2 mL) was centrifuged at 16,000 × *g* at 4°C with a Sorvall RC-5B refrigerated superspeed centrifuge (DuPont Instruments, USA) for 20 min. The pellets were used for total DNA extraction using the FastDNA Spin kit for soil (MP Biomedicals, USA), according to the manufacturer’s protocol. Shotgun metagenomic sequencing was performed using an Illumina HiSeq PE 150 instrument by the commercial sequencing provider Genewiz (Leipzig, Germany). On average, 51.8 × 10^6^ high-quality paired-end reads were generated (see Table S5 in the supplemental material).

10.1128/msystems.00994-22.7 TABLE S5 Numbers of high-quality reads, assembled contigs, and viral contigs that were detected. Download Table S5, DOCX file, 0.02 MB. Copyright © 2023 Wicaksono et al. 2023 Wicaksono et al. https://creativecommons.org/licenses/by/4.0/ This content is distributed under the terms of the Creative Commons Attribution 4.0 International license.

### Read assembly, binning of prokaryotic metagenome-assembled genomes, and viral population recovery.

Default parameters were used for all analysis tools unless otherwise noted. Trimmomatic v0.39 and VSEARCH v2.21.1 were used to remove Illumina sequencing adaptors and perform initial quality filtering (removal of low-quality reads with a Phred score of <20) on the metagenomic reads. Briefly, we assembled high-quality reads using MEGAHIT v1.2.9 with meta-sensitive parameters (49). Only contigs with a length of >10 kb were retained for binning metagenome-assembled genomes (MAGs) and putative viral contigs. Maxbin2 v2.2.7, MetaBAT2 v2.15, and CONCOCT v1.1.0 (50–52) were used to bin MAGs, and DASTool v1.1.4 (53) was implemented to dereplicate the MAGs. The quality of the MAGs was estimated using CheckM v1.2.1 (54), and only medium-quality MAGs according to the current definition of the minimum-information metagenome-assembled genome (MIMAG) standards (55) with at least 75% completeness were retained for further analyses. Taxonomic information for each MAG was obtained using GTDB-Tk v2.1.1 (56). For the identification of putative viral contigs in the metagenome data sets, we used two methods: (i) the nontargeted virus sequence discovery pipeline as described previously (57), based on comparisons of open reading frames of metagenome contigs to a set of 25,281 viral protein families (VPFs) from known viruses, and (ii) VirSorter v1.0.6, a tool to identify viral sequences from complex microbial DNA samples (58). These two analysis strategies are suitable for virus detection directly from microbiome data sets that are generated through untargeted approaches without viral particle enrichment. We kept only contigs that were classified into categories 1 and 2 as well as categories 4 and 5 according to VirSorter for further analyses to avoid nonviral sequences. These contigs likely represent viral and prophage genomes (58). A total of 2,155 viral contigs were identified using both of the above-described approaches (Table S5). The putative viral contigs generated from the two approaches were clustered into nonredundant viral contigs using CD-HIT-EST v4.8.1 at 95% nucleotide identity (59) over 85% of the shorter contig’s length and defined as viral populations (based on vOTUs). CheckV v1.0.1 was used to assess the quality and completeness of vOTUs. We also used BACPHLIP v0.9.6, a computation tool for predicting viral lifestyles based on conserved protein domains (37). Abundances within vOTUs and bacterial genomes were further estimated using coverM v0.6.1.

### Amplicon sequencing of prokaryotic marker genes.

To examine the association between viral and prokaryotic community structures, the DNA samples were subjected to amplicon sequencing. We used primer set 515f/806r to amplify the V4 regions of prokaryotic 16S rRNA genes using PCR parameters described previously (60). The PCR mixture (25 μL) contained 1× Taq&Go (MP Biomedicals, Illkirch, France), 0.25 mM each primer, and 1 μL template DNA. The PCR products were further purified using the Wizard SV gel and PCR cleanup kit (Promega), pooled in equimolar concentrations, and then sequenced using an Illumina MiSeq PE 300 instrument by the sequencing provider Genewiz (Leipzig, Germany).

We used QIIME2 version 2019.10 (https://qiime2.org) (61) to analyze the amplicon sequencing data set. Marker gene primers were trimmed from the raw reads, and the raw reads were further demultiplexed with the cutadapt tool (62). The trimmed reads were then subjected to quality filtering, denoising, and chimeric sequence removal using the DADA2 algorithm (63). The generated amplicon sequence variants (ASVs) were subsequently aligned against the Silva v128 reference database (64) using the VSEARCH classifier (65) to obtain taxonomic information for each ASV.

### Classification and construction of viral clusters via a gene-sharing network of vOTUs.

As there is no known universal marker gene for the taxonomic identification of viruses, a gene-sharing network analysis was implemented. vConTACT2 v0.11.3 was used to cluster vOTUs with relatively high gene content similarities (66). Briefly, putative viral sequences were classified using the BLASTP algorithm implemented in vConTACT2 using the prokaryotic ViralRefSeq 201 database, the Markov clustering algorithm (MCL) for protein clustering (67), and ClusterONE (68) for genome clustering. Subsequently, the network file was visualized with Cytoscape using an edge-weighted spring-embedded layout, which places the genomes sharing more PCs closer to each other (69).

### Reconstruction of virus-host linkages.

To infer putative virus-host links, three different *in silico* methods were used. (i) For host CRISPR spacer matching, CRISPR spacers were searched using MinCED (options -minNR 2 -spacers) (70) with default parameters. The obtained CRISPR spacer sequences were then aligned against the vOTU sequences using BLASTn. A spacer hit was considered positive with 100% coverage, an E value of ≤0.001, and ≤2 mismatches over the complete length. (ii) For nucleotide sequence homology, BLASTn was used to align the sequences of vOTUs and prokaryotic MAGs. The match criteria were ≥75% coverage over the length of the viral contig, ≥70% minimum nucleotide identity, a bit score of ≥50, and an E value of ≤0.001. (iii) For oligonucleotide frequencies (ONFs), VirHostMatcher v1.0.0 was used; it computes various ONFs based on distance/dissimilarity measures between vOTUs and putative host genomes (25). VirHostMatcher was run with default parameters, and $d_{2}^{*}$ values of ≤0.17 were considered a match against a collection of archaeal and bacterial MAGs from the metagenome. This value was used as a threshold because it yields >80% accuracy across all taxonomic levels in predicting putative viral hosts. When more than one host was predicted for a vOTU, the virus-host link was chosen based on the ranking criteria that were reported previously (45), as follows: (i) host CRISPR spacer match, (ii) nucleotide sequence homology using BLASTn, and (iii) best-matching ONF patterns.

### Auxiliary metabolic gene analysis.

DRAM-v v1.3.5 (47) and VIBRANT v1.2.1 (48) were used to perform auxiliary metabolic gene (AMG) analysis. Because DRAM-v requires an output produced by VirSorter2, we subjected all of the detected vOTUs to VirSorter2 v2.2.3 (71) using the –prep-for-dramv parameter to generate the affi-contigs.tab file and then used DRAM-v to perform AMG analysis with the default databases. A gene was considered a candidate AMG if the auxiliary score was <4 and it had an AMG flag of “-M” or “-F.” We then performed a manual inspection of the genomic context. We excluded AMGs that were associated with nucleotide metabolism, organic nitrogen, glycosyltransferases, and ribosomal proteins, as described previously (72). A gene was a high-confidence viral AMG if the gene was located between two viral hallmark genes or virus-like genes or was located next to a viral hallmark gene or a virus-like gene. A genomic map of vOTUs containing AMGs of interest was visualized using the gggenes R package (73). The NCBI CD-search tool (74) was used to identify conserved domains. For selected AMGs, the amino acid sequences of the AMGs were used as the input for the Phyre2 Web portal (75) to search for protein structural homology and predict the three-dimensional structures of viral proteins.

### Statistical analysis.

Statistical analyses were performed in RStudio v1.3.1093 using the Phyloseq, MicrobiomeAnalyst, and vegan R packages (76–81). The nonparametric (rank-based) Kruskal-Wallis test followed by the pairwise Wilcox test was used to statistically examine differences in the alpha diversity values and relative abundances of vOTUs and bacterial genomes between samples. Microbial and viral composition data were used to construct Bray-Curtis and Jaccard dissimilarity matrices and then subjected to PERMANOVA to test for significant effects of factors on the microbial and viral community structures. The Mantel test was used to measure the correlation between two distance matrices, e.g., microbial and viral community dissimilarities.

### Data availability.

The data from this shotgun metagenome project have been deposited in the European Nucleotide Archive (ENA) database under study accession number PRJEB51329.
