# Supplementary material for: Viral Community Structure and Potential Functions in the Dried-Out Aral Sea Basin Change along a Desiccation Gradient
Source: mSystems. 2023 Jan 10;8(1):e00994-22. doi: 10.1128/msystems.00994-22 (PMC9948696; doi:10.1128/msystems.00994-22)
Supplement: TABLE S1 [file msystems.00994-22-s0004.docx]

**Table S1. Details of the recovered viral population (vOTUs)**

| vOTU ID | Contig length | CheckV quality | Taxonomic classification according to vContact2 | | Putative host | Predicted lifestyle |
| --- | --- | --- | --- | --- | --- | --- |
|  |  |  | Order | Family |  |  |
| vOTU1 | 25155 | Not-determined | Unassigned | Unassigned | *Gammaproteobacteria* | Not determined |
| vOTU9 | 35019 | Medium-quality | Caudovirales | Siphoviridae | *Halobacteria* | Not determined |
| vOTU10 | 10988 | Low-quality | Unassigned | Unassigned | *Rhodothermia* | Not determined |
| vOTU15 | 18291 | Not-determined | Unassigned | Unassigned | *Halobacteria* | Lysogeny |
| vOTU16 | 13622 | Low-quality | Unassigned | Unassigned | *Halobacteria* | Not determined |
| vOTU17 | 12327 | High-quality | Unassigned | Unassigned | *Bacilli* | Not determined |
| vOTU18 | 45354 | High-quality | Unassigned | Unassigned | *Gammaproteobacteria* | Lysogeny |
| vOTU23 | 33461 | Low-quality | Caudovirales | Siphoviridae | *Halobacteria* | Not determined |
| vOTU25 | 15877 | Low-quality | Unassigned | Unassigned | *Actinomycetia* | Not determined |
| vOTU26 | 11149 | Medium-quality | Unassigned | Unassigned | *Bacilli* | Not determined |
| vOTU28 | 92644 | Medium-quality | Caudovirales | Myoviridae | *Gammaproteobacteria* | Not determined |
| vOTU29 | 10024 | Low-quality | Caudovirales | Siphoviridae | *Bacilli* | Not determined |
| vOTU34 | 17441 | Low-quality | Unassigned | Unassigned | *Gammaproteobacteria* | Not determined |
| vOTU40 | 11302 | Low-quality | Unassigned | Unassigned | *Gammaproteobacteria* | Not determined |
| vOTU45 | 11915 | Not-determined | Unassigned | Unassigned | *Gammaproteobacteria* | Not determined |
| vOTU46 | 27342 | Low-quality | Unassigned | Unassigned | *Gammaproteobacteria* | Not determined |
| vOTU61 | 40299 | High-quality | Caudovirales | Myoviridae | *Gammaproteobacteria* | Lysogeny |
| vOTU62 | 12831 | Not-determined | Unassigned | Unassigned | *Gammaproteobacteria* | Lysogeny |
| vOTU65 | 16524 | Low-quality | Unassigned | Unassigned | *Gammaproteobacteria* | Lysogeny |
| vOTU66 | 15022 | Low-quality | Unassigned | Unassigned | *Rhodothermia* | Not determined |
| vOTU67 | 10626 | Low-quality | Unassigned | Unassigned | *Actinomycetia* | Lysogeny |
| vOTU69 | 13434 | Low-quality | Unassigned | Unassigned | *Chloroflexia* | Not determined |
| vOTU70 | 55338 | Medium-quality | Unassigned | Unassigned | *Gammaproteobacteria* | Lysogeny |
| vOTU71 | 42785 | High-quality | Unassigned | Unassigned | *Chloroflexia* | Not determined |
| vOTU72 | 50461 | Low-quality | Unassigned | Unassigned | *Bacilli* | Not determined |
| vOTU74 | 12603 | Low-quality | Unassigned | Unassigned | *Bacteroidia* | Lysogeny |
| vOTU76 | 24873 | Low-quality | Unassigned | Unassigned | *Gammaproteobacteria* | Not determined |
| vOTU78 | 10364 | Low-quality | Unassigned | Unassigned | *Gammaproteobacteria* | Not determined |
| vOTU85 | 43080 | Medium-quality | Unassigned | Unassigned | *Gammaproteobacteria* | Lysogeny |
| vOTU87 | 10311 | Low-quality | Unassigned | Unassigned | *Halobacteria* | Not determined |
| vOTU90 | 68329 | High-quality | Caudovirales | Siphoviridae | *Halobacteria* | Lysogeny |
| vOTU94 | 11681 | Low-quality | Unassigned | Unassigned | *Gammaproteobacteria* | Not determined |
| vOTU105 | 12476 | Not-determined | Unassigned | Unassigned | *Rhodothermia* | Not determined |
| vOTU107 | 75325 | Complete | Caudovirales | Podoviridae | *Gammaproteobacteria* | Lysogeny |
| vOTU108 | 19663 | Not-determined | Unassigned | Unassigned | *Halobacteria* | Lysogeny |
| vOTU111 | 43936 | Medium-quality | Unassigned | Unassigned | *Actinomycetia* | Lysogeny |
| vOTU112 | 11372 | Low-quality | Unassigned | Unassigned | *Gammaproteobacteria* | Not determined |
| vOTU114 | 17529 | Low-quality | Unassigned | Unassigned | *Actinomycetia* | Lysogeny |
| vOTU120 | 13625 | Low-quality | Unassigned | Unassigned | *Rhodothermia* | Not determined |
| vOTU125 | 32178 | Medium-quality | Caudovirales | Myoviridae | *Gammaproteobacteria* | Not determined |
| vOTU133 | 19562 | Low-quality | Unassigned | Unassigned | *Halobacteria* | Not determined |
| vOTU138 | 13157 | Not-determined | Unassigned | Unassigned | *Halobacteria* | Lysogeny |
| vOTU142 | 10776 | Not-determined | Unassigned | Unassigned | *Rhodothermia* | Not determined |
| vOTU144 | 16585 | Not-determined | Unassigned | Unassigned | *Rhodothermia* | Not determined |
| vOTU145 | 60834 | Low-quality | Unassigned | Unassigned | *Bacilli* | Lysogeny |
| vOTU149 | 14916 | Low-quality | Unassigned | Unassigned | *Gammaproteobacteria* | Not determined |
| vOTU151 | 39222 | Low-quality | Unassigned | Unassigned | *Gammaproteobacteria* | Not determined |
| vOTU152 | 68624 | Medium-quality | Unassigned | Unassigned | *Gammaproteobacteria* | Lysogeny |
| vOTU154 | 11974 | Not-determined | Haloruvirales | Pleolipoviridae | *Halobacteria* | Lysogeny |
| vOTU156 | 12397 | Not-determined | Unassigned | Unassigned | *Halobacteria* | Lysogeny |
| vOTU157 | 19026 | Low-quality | Unassigned | Unassigned | *Halobacteria* | Not determined |
| vOTU159 | 11610 | Low-quality | Caudovirales | Myoviridae | *Gammaproteobacteria* | Not determined |
| vOTU160 | 40241 | Medium-quality | Unassigned | Unassigned | *Gammaproteobacteria* | Lysogeny |
| vOTU166 | 11563 | Not-determined | Haloruvirales | Pleolipoviridae | *Halobacteria* | Not determined |
| vOTU169 | 10030 | Low-quality | Unassigned | Unassigned | *Bacilli* | Lysogeny |
| vOTU170 | 15151 | Not-determined | Unassigned | Unassigned | *Halobacteria* | Not determined |
| vOTU174 | 11891 | Not-determined | Unassigned | Unassigned | *Gammaproteobacteria* | Not determined |
| vOTU175 | 19149 | Low-quality | Unassigned | Unassigned | *Bacilli* | Lysogeny |
| vOTU176 | 45712 | High-quality | Caudovirales | Myoviridae | *Gammaproteobacteria* | Lysogeny |
| vOTU178 | 13518 | Low-quality | Unassigned | Unassigned | *Rhodothermia* | Lysogeny |
| vOTU181 | 17888 | Low-quality | Unassigned | Unassigned | *Gammaproteobacteria* | Not determined |
| vOTU188 | 12585 | Not-determined | Unassigned | Unassigned | *Halobacteria* | Not determined |
| vOTU192 | 17596 | Low-quality | Unassigned | Unassigned | *Gammaproteobacteria* | Not determined |
| vOTU194 | 167655 | Complete | Unassigned | Unassigned | *Nitrososphaeria* | Lysogeny |
| vOTU195 | 35990 | Not-determined | Unassigned | Unassigned | *Halobacteria* | Lysogeny |
| vOTU196 | 19288 | Low-quality | Unassigned | Halspiviridae | *Halobacteria* | Not determined |
| vOTU202 | 28742 | Low-quality | Unassigned | Unassigned | *Gammaproteobacteria* | Not determined |
| vOTU204 | 18688 | Low-quality | Unassigned | Unassigned | *Gammaproteobacteria* | Not determined |
| vOTU205 | 35686 | Medium-quality | Unassigned | Unassigned | *Rhodothermia* | Lysogeny |
| vOTU206 | 30195 | Low-quality | Caudovirales | Myoviridae | *Gammaproteobacteria* | Not determined |
| vOTU207 | 54922 | Medium-quality | Unassigned | Unassigned | *Gammaproteobacteria* | Lysogeny |
| vOTU210 | 13438 | Low-quality | Unassigned | Unassigned | *Gammaproteobacteria* | Not determined |
| vOTU211 | 35754 | Medium-quality | Unassigned | Unassigned | *Gammaproteobacteria* | Not determined |
| vOTU214 | 14099 | Low-quality | Unassigned | Unassigned | *Gammaproteobacteria* | Lysogeny |
| vOTU218 | 14608 | Low-quality | Unassigned | Unassigned | *Actinomycetia* | Lysogeny |
| vOTU228 | 10249 | Not-determined | Unassigned | Unassigned | *Gammaproteobacteria* | Lysogeny |
| vOTU229 | 30067 | Low-quality | Unassigned | Unassigned | *Gammaproteobacteria* | Lysogeny |
| vOTU230 | 10426 | Not-determined | Caudovirales | Siphoviridae | *Actinomycetia* | Lysogeny |
| vOTU232 | 13960 | Low-quality | Unassigned | Unassigned | *Alphaproteobacteria* | Not determined |
| vOTU234 | 10944 | Low-quality | Unassigned | Unassigned | *Gammaproteobacteria* | Lysogeny |
| vOTU238 | 69006 | Medium-quality | Unassigned | Unassigned | *Actinomycetia* | Lysogeny |
| vOTU256 | 16080 | Low-quality | Unassigned | Unassigned | *Gammaproteobacteria* | Not determined |
| vOTU265 | 14720 | Low-quality | Unassigned | Unassigned | *Gammaproteobacteria* | Not determined |
| vOTU267 | 11000 | Low-quality | Unassigned | Unassigned | *Gammaproteobacteria* | Not determined |
| vOTU268 | 11522 | Not-determined | Unassigned | Unassigned | *Gammaproteobacteria* | Not determined |
| vOTU270 | 17576 | Low-quality | Unassigned | Unassigned | *Alphaproteobacteria* | Not determined |
| vOTU271 | 20140 | Low-quality | Unassigned | Unassigned | *Gammaproteobacteria* | Lysogeny |
| vOTU280 | 19592 | Low-quality | Unassigned | Unassigned | *Gammaproteobacteria* | Lysogeny |
| vOTU286 | 14374 | Low-quality | Unassigned | Unassigned | *Gammaproteobacteria* | Not determined |
| vOTU289 | 22359 | Low-quality | Unassigned | Unassigned | *Gammaproteobacteria* | Not determined |
| vOTU290 | 45237 | Medium-quality | Caudovirales | Myoviridae | *Gammaproteobacteria* | Lysogeny |
| vOTU297 | 14219 | Low-quality | Unassigned | Unassigned | *Rhodothermia* | Not determined |
| vOTU301 | 13394 | Low-quality | Unassigned | Unassigned | *Actinomycetia* | Not determined |
| vOTU302 | 17190 | Low-quality | Unassigned | Unassigned | *Bacteroidia* | Lysogeny |
| vOTU303 | 12130 | Low-quality | Unassigned | Unassigned | *Gammaproteobacteria* | Not determined |
| vOTU309 | 10587 | Low-quality | Unassigned | Unassigned | *Gammaproteobacteria* | Not determined |
| vOTU311 | 21050 | Low-quality | Unassigned | Unassigned | *Gammaproteobacteria* | Lysogeny |
| vOTU314 | 13466 | Low-quality | Unassigned | Unassigned | *Verrucomicrobiae* | Not determined |
| vOTU315 | 38630 | Medium-quality | Unassigned | Unassigned | *Gammaproteobacteria* | Lysogeny |
| vOTU317 | 11256 | Low-quality | Unassigned | Unassigned | *Gammaproteobacteria* | Not determined |
| vOTU321 | 10990 | Low-quality | Unassigned | Unassigned | *Rhodothermia* | Not determined |
| vOTU322 | 11289 | Low-quality | Unassigned | Unassigned | *Gammaproteobacteria* | Not determined |
| vOTU333 | 22458 | Low-quality | Unassigned | Unassigned | *Gammaproteobacteria* | Not determined |
| vOTU336 | 11679 | Not-determined | Unassigned | Unassigned | *Gammaproteobacteria* | Not determined |
| vOTU342 | 11166 | Low-quality | Unassigned | Unassigned | *Gammaproteobacteria* | Not determined |
| vOTU343 | 12785 | Low-quality | Unassigned | Unassigned | *Bacilli* | Lysogeny |
| vOTU344 | 10271 | Low-quality | Unassigned | Unassigned | *Gammaproteobacteria* | Not determined |
| vOTU346 | 59705 | Medium-quality | Unassigned | Unassigned | *Gammaproteobacteria* | Lysogeny |
| vOTU347 | 22964 | Low-quality | Unassigned | Unassigned | *Gammaproteobacteria* | Not determined |
| vOTU348 | 14011 | Low-quality | Unassigned | Unassigned | *Gammaproteobacteria* | Not determined |
| vOTU349 | 63223 | Medium-quality | Unassigned | Unassigned | *Gammaproteobacteria* | Lysogeny |
| vOTU350 | 11303 | Low-quality | Unassigned | Unassigned | *Gammaproteobacteria* | Not determined |
| vOTU352 | 21404 | Low-quality | Unassigned | Unassigned | *Chloroflexia* | Not determined |
| vOTU355 | 10444 | Low-quality | Unassigned | Unassigned | *Gammaproteobacteria* | Lysogeny |
| vOTU356 | 12362 | Low-quality | Unassigned | Unassigned | *Gammaproteobacteria* | Not determined |
| vOTU359 | 12305 | Low-quality | Unassigned | Unassigned | *Gammaproteobacteria* | Not determined |
| vOTU364 | 23922 | Low-quality | Unassigned | Unassigned | *Actinomycetia* | Lysogeny |
| vOTU369 | 19858 | Low-quality | Unassigned | Unassigned | *Rhodothermia* | Not determined |
| vOTU371 | 16748 | Low-quality | Unassigned | Unassigned | *Polyangia* | Not determined |
| vOTU372 | 11597 | Low-quality | Unassigned | Unassigned | *Gammaproteobacteria* | Lysogeny |
| vOTU377 | 10685 | Not-determined | Unassigned | Unassigned | *Gammaproteobacteria* | Lysogeny |
| vOTU380 | 11487 | Low-quality | Unassigned | Unassigned | *Gammaproteobacteria* | Not determined |
| vOTU390 | 20024 | Low-quality | Unassigned | Unassigned | *Gammaproteobacteria* | Not determined |
| vOTU392 | 11388 | Low-quality | Unassigned | Unassigned | *Gammaproteobacteria* | Not determined |
| vOTU395 | 26692 | Medium-quality | Unassigned | Unassigned | *Alphaproteobacteria* | Not determined |
| vOTU418 | 10949 | Low-quality | Unassigned | Unassigned | *Rhodothermia* | Not determined |
| vOTU419 | 16930 | Low-quality | Unassigned | Unassigned | *Gammaproteobacteria* | Not determined |
| vOTU423 | 11515 | Low-quality | Unassigned | Unassigned | *Gammaproteobacteria* | Not determined |
| vOTU428 | 10608 | Low-quality | Unassigned | Unassigned | *Gammaproteobacteria* | Not determined |
| vOTU434 | 14942 | Low-quality | Unassigned | Unassigned | *Bacilli* | Not determined |
| vOTU444 | 12455 | Low-quality | Unassigned | Unassigned | *Gammaproteobacteria* | Not determined |
| vOTU448 | 13347 | Low-quality | Unassigned | Unassigned | *Gammaproteobacteria* | Lysogeny |
| vOTU453 | 10118 | Not-determined | Unassigned | Unassigned | *Rhodothermia* | Lysogeny |
| vOTU455 | 49861 | Low-quality | Unassigned | Unassigned | *Gammaproteobacteria* | Lysogeny |
| vOTU456 | 12559 | Low-quality | Unassigned | Unassigned | *Gammaproteobacteria* | Not determined |
| vOTU459 | 29313 | Not-determined | Unassigned | Unassigned | *Rhodothermia* | Lysogeny |
| vOTU475 | 14616 | Low-quality | Unassigned | Unassigned | *SAR324* | Lysogeny |
| vOTU480 | 13200 | Low-quality | Unassigned | Unassigned | *Gammaproteobacteria* | Not determined |
| vOTU489 | 11803 | Not-determined | Unassigned | Unassigned | *Gemmatimonadetes* | Not determined |
| vOTU496 | 16331 | Not-determined | Unassigned | Unassigned | *Gammaproteobacteria* | Lysogeny |
| vOTU497 | 13983 | Low-quality | Unassigned | Unassigned | *Actinomycetia* | Not determined |
| vOTU500 | 11603 | Low-quality | Unassigned | Unassigned | *Actinomycetia* | Not determined |
| vOTU503 | 11906 | Low-quality | Unassigned | Unassigned | *Actinomycetia* | Not determined |
| vOTU506 | 10667 | Low-quality | Unassigned | Unassigned | *Bacteroidia* | Not determined |
| vOTU507 | 15876 | Low-quality | Unassigned | Unassigned | *Polyangia* | Not determined |
| vOTU512 | 10261 | Low-quality | Unassigned | Unassigned | *Bacteroidia* | Not determined |
| vOTU518 | 10630 | Low-quality | Unassigned | Unassigned | *Gammaproteobacteria* | Not determined |
| vOTU522 | 11927 | Low-quality | Unassigned | Unassigned | *Rhodothermia* | Not determined |
| vOTU530 | 10413 | Medium-quality | Unassigned | Unassigned | *Alphaproteobacteria* | Not determined |
| vOTU531 | 14599 | Low-quality | Unassigned | Unassigned | *Gammaproteobacteria* | Not determined |
| vOTU548 | 10428 | Low-quality | Unassigned | Unassigned | *Rhodothermia* | Not determined |
| vOTU551 | 312851 | High-quality | Unassigned | Unassigned | *Gammaproteobacteria* | Lysogeny |
| vOTU559 | 11941 | Low-quality | Unassigned | Unassigned | *Alphaproteobacteria* | Lysogeny |
| vOTU563 | 12855 | Low-quality | Unassigned | Unassigned | *Bacteroidia* | Not determined |
| vOTU575 | 11649 | Low-quality | Unassigned | Unassigned | *Chloroflexia* | Not determined |
| vOTU578 | 14254 | Low-quality | Unassigned | Unassigned | *Halobacteria* | Not determined |
| vOTU581 | 12657 | Low-quality | Unassigned | Unassigned | *Gammaproteobacteria* | Not determined |
| vOTU584 | 11992 | Low-quality | Unassigned | Unassigned | *Gammaproteobacteria* | Lysogeny |
| vOTU588 | 44520 | Low-quality | Unassigned | Unassigned | *Halobacteria* | Lysogeny |
| vOTU590 | 11574 | Low-quality | Unassigned | Unassigned | *Gammaproteobacteria* | Not determined |
| vOTU591 | 14256 | Low-quality | Unassigned | Unassigned | *Gammaproteobacteria* | Lysogeny |
| vOTU592 | 38706 | Medium-quality | Caudovirales | Myoviridae | *Gammaproteobacteria* | Lysogeny |
| vOTU593 | 19787 | Low-quality | Unassigned | Unassigned | *Gammaproteobacteria* | Lysogeny |
| vOTU594 | 13838 | Low-quality | Unassigned | Unassigned | *Gammaproteobacteria* | Not determined |
| vOTU595 | 11154 | Low-quality | Unassigned | Unassigned | *Rhodothermia* | Lysogeny |
| vOTU598 | 11649 | Not-determined | Unassigned | Unassigned | *Thermoanaerobaculia* | Not determined |
| vOTU601 | 14247 | Low-quality | Unassigned | Unassigned | *Polyangia* | Not determined |
| vOTU602 | 60413 | Low-quality | Unassigned | Unassigned | *Gammaproteobacteria* | Lysogeny |
| vOTU603 | 11465 | Low-quality | Unassigned | Unassigned | *Gammaproteobacteria* | Not determined |
| vOTU608 | 49320 | Low-quality | Unassigned | Unassigned | *Gammaproteobacteria* | Not determined |
| vOTU609 | 38578 | Medium-quality | Unassigned | Unassigned | *Gammaproteobacteria* | Lysogeny |
| vOTU610 | 22071 | Low-quality | Caudovirales | Siphoviridae | *Alphaproteobacteria* | Not determined |
| vOTU616 | 16354 | Low-quality | Unassigned | Unassigned | *Gammaproteobacteria* | Not determined |
| vOTU619 | 11145 | Not-determined | Unassigned | Unassigned | *Gammaproteobacteria* | Not determined |
| vOTU622 | 11634 | Low-quality | Unassigned | Unassigned | *Gammaproteobacteria* | Not determined |
| vOTU625 | 12154 | Low-quality | Unassigned | Unassigned | *Bacilli* | Lysogeny |
| vOTU627 | 11027 | Low-quality | Unassigned | Unassigned | *Gammaproteobacteria* | Lysogeny |
| vOTU634 | 14610 | Low-quality | Unassigned | Unassigned | *Actinomycetia* | Not determined |
| vOTU646 | 12762 | Not-determined | Unassigned | Unassigned | *Gammaproteobacteria* | Lysogeny |
| vOTU652 | 12164 | Low-quality | Unassigned | Unassigned | *Gammaproteobacteria* | Not determined |
| vOTU657 | 11769 | Low-quality | Unassigned | Unassigned | *Gammaproteobacteria* | Lysogeny |
| vOTU661 | 10599 | Low-quality | Unassigned | Unassigned | *Actinomycetia* | Not determined |
| vOTU666 | 14725 | Low-quality | Unassigned | Unassigned | *Gammaproteobacteria* | Lysogeny |
| vOTU669 | 20679 | Low-quality | Unassigned | Unassigned | *Gammaproteobacteria* | Lysogeny |
| vOTU672 | 10129 | Not-determined | Unassigned | Unassigned | *Actinomycetia* | Not determined |
| vOTU680 | 13597 | Low-quality | Unassigned | Unassigned | *Actinomycetia* | Lysogeny |
| vOTU689 | 34850 | Low-quality | Unassigned | Unassigned | *Bacteroidia* | Not determined |
| vOTU695 | 10089 | Low-quality | Unassigned | Unassigned | *Rhodothermia* | Not determined |
| vOTU698 | 29682 | Low-quality | Unassigned | Unassigned | *Gammaproteobacteria* | Not determined |
| vOTU702 | 28553 | Low-quality | Unassigned | Unassigned | *Gammaproteobacteria* | Lysogeny |
| vOTU706 | 25523 | Low-quality | Unassigned | Unassigned | *Actinomycetia* | Not determined |
| vOTU710 | 11822 | Not-determined | Unassigned | Unassigned | *Bacteroidia* | Not determined |
| vOTU716 | 14924 | Low-quality | Unassigned | Unassigned | *Polyangia* | Not determined |
| vOTU718 | 13622 | Not-determined | Unassigned | Unassigned | *Polyangia* | Lysogeny |
| vOTU723 | 78839 | Medium-quality | Unassigned | Unassigned | *Gammaproteobacteria* | Lysogeny |
| vOTU724 | 17409 | Not-determined | Unassigned | Unassigned | *Thermoanaerobaculia* | Not determined |
| vOTU730 | 24935 | Medium-quality | Unassigned | Unassigned | *Gammaproteobacteria* | Not determined |
| vOTU735 | 24001 | Low-quality | Unassigned | Unassigned | *Gammaproteobacteria* | Not determined |
| vOTU743 | 12220 | Not-determined | Unassigned | Unassigned | *Actinomycetia* | Not determined |
| vOTU744 | 29132 | Low-quality | Unassigned | Unassigned | *Actinomycetia* | Not determined |
| vOTU745 | 36032 | Medium-quality | Unassigned | Unassigned | *Gammaproteobacteria* | Not determined |
| vOTU748 | 19150 | Low-quality | Unassigned | Unassigned | *Actinomycetia* | Not determined |
| vOTU752 | 11916 | Low-quality | Unassigned | Unassigned | *Actinomycetia* | Not determined |
| vOTU757 | 41562 | Medium-quality | Unassigned | Unassigned | *Actinomycetia* | Lysogeny |
| vOTU758 | 45338 | Medium-quality | Unassigned | Unassigned | *Actinomycetia* | Lysogeny |
| vOTU759 | 30490 | Low-quality | Unassigned | Unassigned | *Verrucomicrobiae* | Lysogeny |
| vOTU760 | 13222 | Low-quality | Unassigned | Unassigned | *Actinomycetia* | Not determined |
| vOTU761 | 74715 | Medium-quality | Unassigned | Unassigned | *Alphaproteobacteria* | Lysogeny |
| vOTU767 | 27700 | Low-quality | Unassigned | Unassigned | *Actinomycetia* | Lysogeny |
| vOTU771 | 55134 | Medium-quality | Unassigned | Unassigned | *Gammaproteobacteria* | Lysogeny |
| vOTU774 | 13554 | Not-determined | Unassigned | Unassigned | *Actinomycetia* | Not determined |
| vOTU776 | 21510 | Not-determined | Unassigned | Unassigned | *Actinomycetia* | Not determined |
| vOTU777 | 79883 | Medium-quality | Unassigned | Unassigned | *Gammaproteobacteria* | Lysogeny |
| vOTU779 | 40411 | Medium-quality | Unassigned | Unassigned | *Gammaproteobacteria* | Lysogeny |
| vOTU782 | 19699 | Low-quality | Unassigned | Unassigned | *Gammaproteobacteria* | Not determined |
| vOTU783 | 12104 | Not-determined | Unassigned | Unassigned | *Gammaproteobacteria* | Not determined |
| vOTU786 | 10653 | Low-quality | Unassigned | Unassigned | *Gammaproteobacteria* | Not determined |
| vOTU797 | 41341 | Low-quality | Unassigned | Unassigned | *Bacteroidia* | Not determined |
| vOTU809 | 13857 | Low-quality | Unassigned | Unassigned | *Bacilli* | Lysogeny |
| vOTU812 | 22529 | Low-quality | Unassigned | Unassigned | *Gammaproteobacteria* | Lysogeny |
| vOTU813 | 22002 | Low-quality | Unassigned | Unassigned | *Gammaproteobacteria* | Lysogeny |
| vOTU818 | 23882 | Low-quality | Unassigned | Unassigned | *Gammaproteobacteria* | Lysogeny |
| vOTU822 | 12086 | Low-quality | Unassigned | Unassigned | *Gammaproteobacteria* | Lysogeny |
| vOTU828 | 20010 | Low-quality | Unassigned | Unassigned | *Actinomycetia* | Not determined |
| vOTU834 | 10463 | Low-quality | Unassigned | Unassigned | *Bacilli* | Not determined |
| vOTU848 | 23720 | Low-quality | Caudovirales | Myoviridae | *Gammaproteobacteria* | Not determined |
| vOTU853 | 10454 | Not-determined | Unassigned | Unassigned | *Actinomycetia* | Not determined |
| vOTU860 | 22104 | Low-quality | Unassigned | Unassigned | *Gammaproteobacteria* | Lysogeny |
| vOTU866 | 10445 | Low-quality | Unassigned | Unassigned | *Gammaproteobacteria* | Not determined |
| vOTU873 | 15840 | Low-quality | Unassigned | Unassigned | *Alphaproteobacteria* | Not determined |
| vOTU887 | 10114 | Low-quality | Unassigned | Unassigned | *Bacilli* | Lysogeny |
| vOTU893 | 12371 | Low-quality | Unassigned | Unassigned | *Gammaproteobacteria* | Not determined |
| vOTU894 | 14010 | Low-quality | Unassigned | Unassigned | *Alphaproteobacteria* | Not determined |
| vOTU896 | 17202 | Low-quality | Unassigned | Halspiviridae | *Gammaproteobacteria* | Lysogeny |
| vOTU897 | 11114 | Low-quality | Unassigned | Unassigned | *Bacteroidia* | Not determined |
| vOTU898 | 70581 | Medium-quality | Caudovirales | Myoviridae | *Gammaproteobacteria* | Lysogeny |
| vOTU899 | 17720 | Low-quality | Unassigned | Unassigned | *Gammaproteobacteria* | Not determined |
| vOTU900 | 100694 | Medium-quality | Unassigned | Unassigned | *Gammaproteobacteria* | Lysogeny |
| vOTU901 | 11858 | Low-quality | Unassigned | Unassigned | *Gammaproteobacteria* | Lysogeny |
| vOTU902 | 31523 | Medium-quality | Unassigned | Unassigned | *Bacilli* | Not determined |
| vOTU903 | 22025 | Low-quality | Unassigned | Unassigned | *Actinomycetia* | Not determined |
| vOTU904 | 21453 | Low-quality | Unassigned | Unassigned | *Gammaproteobacteria* | Not determined |
| vOTU905 | 25836 | Low-quality | Unassigned | Unassigned | *Gammaproteobacteria* | Lysogeny |
| vOTU913 | 11903 | Low-quality | Unassigned | Unassigned | *Alphaproteobacteria* | Not determined |
| vOTU917 | 10575 | Low-quality | Unassigned | Unassigned | *Gammaproteobacteria* | Not determined |
| vOTU918 | 10440 | Low-quality | Unassigned | Unassigned | *Gammaproteobacteria* | Not determined |
| vOTU922 | 11068 | Low-quality | Unassigned | Unassigned | *Gammaproteobacteria* | Lysogeny |
| vOTU926 | 15247 | Low-quality | Unassigned | Unassigned | *Bacilli* | Lysogeny |
| vOTU931 | 12101 | Not-determined | Unassigned | Unassigned | *Gammaproteobacteria* | Not determined |
| vOTU947 | 82832 | Medium-quality | Unassigned | Unassigned | *Gammaproteobacteria* | Not determined |
| vOTU954 | 10031 | Low-quality | Unassigned | Unassigned | *Bacteroidia* | Not determined |
| vOTU955 | 13466 | Low-quality | Unassigned | Unassigned | *Gammaproteobacteria* | Not determined |
| vOTU956 | 12024 | Low-quality | Unassigned | Unassigned | *Gammaproteobacteria* | Lysogeny |
| vOTU969 | 10554 | Low-quality | Unassigned | Unassigned | *Gammaproteobacteria* | Not determined |
| vOTU974 | 10255 | Not-determined | Unassigned | Unassigned | *Chloroflexia* | Not determined |
| vOTU979 | 12773 | Low-quality | Unassigned | Unassigned | *Chloroflexia* | Not determined |
| vOTU991 | 15783 | Low-quality | Unassigned | Unassigned | *Gammaproteobacteria* | Lysogeny |
| vOTU1009 | 10372 | Not-determined | Unassigned | Unassigned | *Alphaproteobacteria* | Lysogeny |
| vOTU1017 | 36962 | Not-determined | Unassigned | Unassigned | *Actinomycetia* | Not determined |
| vOTU1019 | 27799 | Low-quality | Unassigned | Unassigned | *Actinomycetia* | Lysogeny |
| vOTU1025 | 15742 | Low-quality | Unassigned | Unassigned | *Actinomycetia* | Not determined |
| vOTU1026 | 15518 | Low-quality | Unassigned | Unassigned | *Bacilli* | Lysogeny |
| vOTU1029 | 11604 | Not-determined | Unassigned | Unassigned | *Gammaproteobacteria* | Not determined |
| vOTU1032 | 14258 | Low-quality | Unassigned | Unassigned | *Actinomycetia* | Not determined |
| vOTU1033 | 18298 | Low-quality | Unassigned | Unassigned | *Gammaproteobacteria* | Not determined |
| vOTU1037 | 34615 | Low-quality | Unassigned | Unassigned | *Bacilli* | Lysogeny |
| vOTU1040 | 14303 | Low-quality | Unassigned | Unassigned | *Gammaproteobacteria* | Not determined |
| vOTU1045 | 10805 | Low-quality | Unassigned | Unassigned | *Gammaproteobacteria* | Not determined |
| vOTU1048 | 10147 | Low-quality | Unassigned | Unassigned | *Bacteroidia* | Not determined |
| vOTU1057 | 11273 | Low-quality | Unassigned | Unassigned | *Gammaproteobacteria* | Not determined |
| vOTU1058 | 34093 | Low-quality | Unassigned | Unassigned | *Actinomycetia* | Not determined |
| vOTU1059 | 13961 | Not-determined | Unassigned | Unassigned | *Rhodothermia* | Not determined |
| vOTU1060 | 12086 | Low-quality | Unassigned | Unassigned | *Actinomycetia* | Lysogeny |
| vOTU1061 | 37359 | Low-quality | Unassigned | Unassigned | *Gammaproteobacteria* | Not determined |
| vOTU1062 | 17559 | Low-quality | Unassigned | Unassigned | *Actinomycetia* | Lysogeny |
| vOTU1063 | 36662 | Medium-quality | Unassigned | Unassigned | *Gammaproteobacteria* | Not determined |
| vOTU1064 | 18311 | Low-quality | Unassigned | Unassigned | *Gammaproteobacteria* | Not determined |
| vOTU1065 | 24378 | Low-quality | Unassigned | Unassigned | *Gammaproteobacteria* | Lysogeny |
| vOTU1072 | 34096 | Low-quality | Unassigned | Unassigned | *Actinomycetia* | Not determined |
| vOTU1075 | 51633 | Low-quality | Unassigned | Unassigned | *Bacilli* | Not determined |
| vOTU1089 | 11295 | Low-quality | Unassigned | Unassigned | *Bacteroidia* | Not determined |
| vOTU1092 | 10628 | Low-quality | Unassigned | Unassigned | *Gammaproteobacteria* | Not determined |
| vOTU1097 | 11906 | Low-quality | Unassigned | Unassigned | *Rhodothermia* | Not determined |
| vOTU1103 | 11691 | Low-quality | Unassigned | Unassigned | *Actinomycetia* | Lysogeny |
| vOTU1111 | 10710 | Low-quality | Unassigned | Unassigned | *Bacilli* | Not determined |
| vOTU1112 | 127835 | Medium-quality | Unassigned | Unassigned | *Bacilli* | Not determined |
| vOTU1135 | 26245 | Low-quality | Unassigned | Unassigned | *Halobacteria* | Not determined |
| vOTU1142 | 10674 | Low-quality | Unassigned | Unassigned | *Gemmatimonadetes* | Not determined |
| vOTU1143 | 11257 | Not-determined | Unassigned | Unassigned | *Bacilli* | Not determined |
| vOTU1146 | 10299 | Low-quality | Unassigned | Unassigned | *Gammaproteobacteria* | Not determined |
| vOTU1147 | 18186 | Low-quality | Unassigned | Unassigned | *Bacilli* | Not determined |
| vOTU1148 | 16319 | Low-quality | Unassigned | Unassigned | *Alphaproteobacteria* | Not determined |
| vOTU1152 | 28679 | Low-quality | Unassigned | Unassigned | *Bacilli* | Not determined |
| vOTU1153 | 20672 | Low-quality | Unassigned | Unassigned | *Gammaproteobacteria* | Not determined |
| vOTU1154 | 66853 | Medium-quality | Unassigned | Unassigned | *Gammaproteobacteria* | Lysogeny |
| vOTU1155 | 64958 | Medium-quality | Unassigned | Unassigned | *Gammaproteobacteria* | Lysogeny |
| vOTU1156 | 154400 | Low-quality | Unassigned | Unassigned | *Bacilli* | Not determined |
| vOTU1157 | 59589 | Medium-quality | Caudovirales | Myoviridae | *Gammaproteobacteria* | Lysogeny |
| vOTU1158 | 12641 | Low-quality | Unassigned | Unassigned | *Actinomycetia* | Lysogeny |
| vOTU1165 | 11196 | Low-quality | Unassigned | Unassigned | *Actinomycetia* | Not determined |
| vOTU1167 | 11573 | Low-quality | Unassigned | Unassigned | *Gammaproteobacteria* | Not determined |
| vOTU1176 | 10819 | Low-quality | Unassigned | Unassigned | *Actinomycetia* | Not determined |
| vOTU1184 | 13292 | Low-quality | Unassigned | Unassigned | *Actinomycetia* | Not determined |
| vOTU1188 | 10403 | Not-determined | Unassigned | Unassigned | *Bacteroidia* | Not determined |
| vOTU1195 | 10413 | Low-quality | Unassigned | Unassigned | *Actinomycetia* | Not determined |
| vOTU1197 | 21729 | Low-quality | Unassigned | Unassigned | *Gammaproteobacteria* | Lysogeny |
| vOTU1199 | 59518 | Medium-quality | Unassigned | Unassigned | *Gammaproteobacteria* | Lysogeny |
| vOTU1200 | 12859 | Low-quality | Unassigned | Unassigned | *Actinomycetia* | Not determined |
| vOTU1208 | 10936 | Low-quality | Unassigned | Unassigned | *Gammaproteobacteria* | Not determined |
| vOTU1218 | 12535 | Low-quality | Unassigned | Unassigned | *Gammaproteobacteria* | Not determined |
| vOTU1221 | 10746 | Low-quality | Unassigned | Unassigned | *Gammaproteobacteria* | Lysogeny |
| vOTU1228 | 20119 | Low-quality | Unassigned | Unassigned | *Actinomycetia* | Not determined |
| vOTU1231 | 81497 | Medium-quality | Caudovirales | Siphoviridae | *Alphaproteobacteria* | Lysogeny |
| vOTU1234 | 26948 | Low-quality | Unassigned | Unassigned | *Actinomycetia* | Not determined |
| vOTU1242 | 12705 | Low-quality | Unassigned | Unassigned | *Polyangia* | Not determined |
| vOTU1247 | 16641 | Low-quality | Unassigned | Unassigned | *Rhodothermia* | Not determined |
| vOTU1250 | 12614 | Low-quality | Unassigned | Unassigned | *Actinomycetia* | Not determined |
| vOTU1254 | 39102 | Medium-quality | Unassigned | Unassigned | *Rhodothermia* | Lysogeny |
| vOTU1261 | 14202 | Low-quality | Unassigned | Unassigned | *Thermoleophilia* | Not determined |
| vOTU1265 | 14969 | Low-quality | Unassigned | Unassigned | *Rhodothermia* | Not determined |
| vOTU1270 | 11218 | Low-quality | Unassigned | Unassigned | *Actinomycetia* | Not determined |
| vOTU1273 | 14667 | Not-determined | Unassigned | Unassigned | *Bacteroidia* | Not determined |
| vOTU1274 | 10220 | Low-quality | Unassigned | Unassigned | *Actinomycetia* | Lysogeny |
| vOTU1277 | 10145 | Low-quality | Unassigned | Unassigned | *Chloroflexia* | Not determined |
| vOTU1279 | 75911 | Medium-quality | Unassigned | Unassigned | *Gammaproteobacteria* | Lysogeny |
| vOTU1288 | 17268 | Low-quality | Unassigned | Unassigned | *Actinomycetia* | Not determined |
| vOTU1292 | 10695 | Low-quality | Unassigned | Unassigned | *Chloroflexia* | Not determined |
| vOTU1294 | 15986 | Low-quality | Unassigned | Unassigned | *Bacteroidia* | Not determined |
| vOTU1297 | 13786 | Low-quality | Unassigned | Unassigned | *Actinomycetia* | Lysogeny |
| vOTU1298 | 30980 | Low-quality | Unassigned | Unassigned | *Actinomycetia* | Not determined |
| vOTU1299 | 40092 | Medium-quality | Unassigned | Unassigned | *Gammaproteobacteria* | Lysogeny |
| vOTU1300 | 31598 | Low-quality | Unassigned | Unassigned | *Gammaproteobacteria* | Lysogeny |
| vOTU1303 | 11300 | Low-quality | Unassigned | Unassigned | *Gammaproteobacteria* | Not determined |
| vOTU1305 | 12537 | Low-quality | Unassigned | Unassigned | *Gammaproteobacteria* | Not determined |
| vOTU1317 | 10734 | Low-quality | Unassigned | Unassigned | *Bacilli* | Not determined |
| vOTU1320 | 16487 | Low-quality | Unassigned | Unassigned | *Polyangia* | Not determined |
| vOTU1332 | 10988 | Not-determined | Unassigned | Unassigned | *Actinomycetia* | Not determined |
| vOTU1335 | 14537 | Low-quality | Unassigned | Unassigned | *Bacilli* | Not determined |
| vOTU1340 | 18473 | Low-quality | Unassigned | Unassigned | *Gammaproteobacteria* | Not determined |
| vOTU1341 | 58481 | Low-quality | Unassigned | Unassigned | *Gammaproteobacteria* | Lysogeny |
| vOTU1347 | 13784 | Low-quality | Unassigned | Unassigned | *Bacteroidia* | Not determined |
| vOTU1350 | 49325 | Medium-quality | Unassigned | Unassigned | *Thermoanaerobaculia* | Not determined |
| vOTU1353 | 28205 | Low-quality | Unassigned | Unassigned | *Actinomycetia* | Not determined |
| vOTU1355 | 11620 | Low-quality | Unassigned | Unassigned | *Rhodothermia* | Not determined |
| vOTU1363 | 16668 | Low-quality | Unassigned | Unassigned | *Actinomycetia* | Not determined |
| vOTU1365 | 10003 | Low-quality | Unassigned | Unassigned | *Bacteroidia* | Not determined |
| vOTU1368 | 50211 | Medium-quality | Unassigned | Unassigned | *Actinomycetia* | Not determined |
| vOTU1372 | 10477 | Low-quality | Unassigned | Unassigned | *Gammaproteobacteria* | Not determined |
| vOTU1374 | 13214 | Low-quality | Unassigned | Unassigned | *Actinomycetia* | Not determined |
| vOTU1376 | 10932 | Low-quality | Unassigned | Unassigned | *Bacilli* | Not determined |
| vOTU1388 | 19604 | Low-quality | Unassigned | Unassigned | *Actinomycetia* | Not determined |
| vOTU1398 | 14532 | Low-quality | Unassigned | Unassigned | *Rhodothermia* | Not determined |
| vOTU1401 | 10941 | Low-quality | Unassigned | Unassigned | *Gemmatimonadetes* | Not determined |
| vOTU1404 | 10453 | Low-quality | Unassigned | Unassigned | *Actinomycetia* | Not determined |
| vOTU1407 | 61614 | Medium-quality | Unassigned | Unassigned | *Gammaproteobacteria* | Not determined |
| vOTU1413 | 15194 | Low-quality | Unassigned | Unassigned | *Actinomycetia* | Not determined |
| vOTU1415 | 22990 | Low-quality | Unassigned | Unassigned | *Bacilli* | Not determined |
| vOTU1417 | 11571 | Low-quality | Unassigned | Unassigned | *Gammaproteobacteria* | Not determined |
| vOTU1418 | 15459 | Low-quality | Unassigned | Unassigned | *Gammaproteobacteria* | Not determined |
| vOTU1424 | 10251 | Not-determined | Unassigned | Unassigned | *Actinomycetia* | Not determined |
| vOTU1440 | 16740 | Low-quality | Unassigned | Unassigned | *Actinomycetia* | Not determined |
| vOTU1446 | 12180 | Low-quality | Unassigned | Unassigned | *Actinomycetia* | Not determined |
| vOTU1454 | 11274 | Low-quality | Unassigned | Unassigned | *SAR324* | Not determined |
| vOTU1456 | 29664 | Not-determined | Unassigned | Unassigned | *Gammaproteobacteria* | Not determined |
| vOTU1470 | 13308 | Low-quality | Unassigned | Unassigned | *Actinomycetia* | Not determined |
| vOTU1476 | 15794 | Low-quality | Unassigned | Unassigned | *Actinomycetia* | Not determined |
| vOTU1477 | 16836 | Not-determined | Unassigned | Unassigned | *Actinomycetia* | Not determined |
| vOTU1487 | 15317 | Not-determined | Unassigned | Unassigned | *Gammaproteobacteria* | Not determined |
| vOTU1495 | 10501 | Low-quality | Unassigned | Unassigned | *Actinomycetia* | Not determined |
| vOTU1500 | 19291 | Low-quality | Unassigned | Unassigned | *Rhodothermia* | Not determined |
| vOTU1502 | 78097 | Medium-quality | Unassigned | Unassigned | *Rhodothermia* | Not determined |
| vOTU1503 | 14750 | Low-quality | Unassigned | Unassigned | *Bacilli* | Not determined |
| vOTU1504 | 112994 | High-quality | Unassigned | Unassigned | *Thermoleophilia* | Lysogeny |
| vOTU1505 | 10227 | Low-quality | Unassigned | Unassigned | *Sumerlaeia* | Not determined |
| vOTU1510 | 14779 | Low-quality | Unassigned | Unassigned | *Actinomycetia* | Not determined |
| vOTU1511 | 106555 | Complete | Caudovirales | Myoviridae | *Bacilli* | Lysogeny |
| vOTU1512 | 13525 | Not-determined | Unassigned | Unassigned | *Bacilli* | Not determined |
| vOTU1513 | 18047 | Not-determined | Haloruvirales | Pleolipoviridae | *Halobacteria* | Lysogeny |
| vOTU1514 | 18507 | Not-determined | Haloruvirales | Pleolipoviridae | *Halobacteria* | Lysogeny |
| vOTU1515 | 12321 | Low-quality | Unassigned | Unassigned | *Gammaproteobacteria* | Not determined |
| vOTU1516 | 12843 | Low-quality | Unassigned | Unassigned | *Bacteroidia* | Not determined |
| vOTU1519 | 12151 | Low-quality | Caudovirales | Myoviridae | *Gammaproteobacteria* | Not determined |
| vOTU1520 | 16490 | Low-quality | Unassigned | Unassigned | *Bacteroidia* | Not determined |
| vOTU1521 | 27357 | Low-quality | Unassigned | Unassigned | *Gammaproteobacteria* | Not determined |
| vOTU1522 | 27016 | Low-quality | Unassigned | Unassigned | *Gammaproteobacteria* | Not determined |
| vOTU1524 | 31287 | Low-quality | Unassigned | Unassigned | *Actinomycetia* | Not determined |
| vOTU1525 | 43086 | Medium-quality | Unassigned | Unassigned | *Actinomycetia* | Not determined |
| vOTU1526 | 16028 | Low-quality | Unassigned | Unassigned | *Gammaproteobacteria* | Not determined |
| vOTU1533 | 20344 | Low-quality | Unassigned | Unassigned | *Gammaproteobacteria* | Lysogeny |
| vOTU1541 | 15156 | Low-quality | Unassigned | Unassigned | *Actinomycetia* | Not determined |
| vOTU1542 | 16857 | Low-quality | Unassigned | Unassigned | *Gammaproteobacteria* | Not determined |
| vOTU1544 | 11988 | Low-quality | Caudovirales | Myoviridae | *Gammaproteobacteria* | Not determined |
| vOTU1546 | 52629 | Medium-quality | Unassigned | Unassigned | *Gemmatimonadetes* | Not determined |
| vOTU1548 | 76390 | High-quality | Caudovirales | Podoviridae | *Gammaproteobacteria* | Lysogeny |
| vOTU1550 | 29535 | Medium-quality | Caudovirales | Podoviridae | *Bacteroidia* | Not determined |
| vOTU1553 | 12876 | Low-quality | Unassigned | Unassigned | *Gammaproteobacteria* | Not determined |
| vOTU1554 | 62805 | Medium-quality | Unassigned | Unassigned | *Verrucomicrobiae* | Lysogeny |
| vOTU1555 | 46310 | Medium-quality | Unassigned | Unassigned | *Gammaproteobacteria* | Not determined |
| vOTU1556 | 14452 | Low-quality | Unassigned | Unassigned | *Gammaproteobacteria* | Not determined |
| vOTU1557 | 21528 | Low-quality | Unassigned | Unassigned | *Bacilli* | Not determined |
| vOTU1560 | 73317 | Medium-quality | Unassigned | Unassigned | *Rhodothermia* | Not determined |
| vOTU1561 | 10126 | Low-quality | Unassigned | Unassigned | *Actinomycetia* | Not determined |
| vOTU1562 | 33110 | Low-quality | Unassigned | Unassigned | *Actinomycetia* | Not determined |
| vOTU1563 | 37542 | Medium-quality | Unassigned | Unassigned | *Gammaproteobacteria* | Not determined |
| vOTU1566 | 35381 | Medium-quality | Unassigned | Unassigned | *Gammaproteobacteria* | Not determined |
| vOTU1567 | 48758 | Medium-quality | Unassigned | Unassigned | *Gammaproteobacteria* | Lysogeny |
| vOTU1570 | 25543 | Low-quality | Unassigned | Unassigned | *Polyangia* | Not determined |
| vOTU1571 | 37703 | Medium-quality | Unassigned | Unassigned | *Gammaproteobacteria* | Not determined |
| vOTU1572 | 67115 | Medium-quality | Unassigned | Unassigned | *Polyangia* | Not determined |
| vOTU1573 | 12105 | Low-quality | Unassigned | Unassigned | *Gammaproteobacteria* | Not determined |
| vOTU1574 | 35053 | Medium-quality | Unassigned | Unassigned | *Actinomycetia* | Not determined |
| vOTU1575 | 22545 | Low-quality | Unassigned | Unassigned | *Bacteroidia* | Not determined |
| vOTU1576 | 43954 | Medium-quality | Unassigned | Unassigned | *Gemmatimonadetes* | Not determined |
| vOTU1579 | 24516 | Low-quality | Unassigned | Unassigned | *Gammaproteobacteria* | Not determined |
| vOTU1580 | 14480 | Low-quality | Unassigned | Unassigned | *Gammaproteobacteria* | Not determined |
| vOTU1581 | 61908 | Medium-quality | Caudovirales | Siphoviridae | *Rhodothermia* | Lysogeny |
| vOTU1583 | 38936 | Medium-quality | Unassigned | Unassigned | *Gammaproteobacteria* | Not determined |
| vOTU1584 | 21524 | Low-quality | Unassigned | Unassigned | *Verrucomicrobiae* | Not determined |
| vOTU1585 | 19293 | Low-quality | Unassigned | Unassigned | *Gammaproteobacteria* | Not determined |
| vOTU1588 | 53278 | Medium-quality | Unassigned | Unassigned | *Rhodothermia* | Not determined |
| vOTU1591 | 32454 | Low-quality | Unassigned | Unassigned | *Gammaproteobacteria* | Not determined |
| vOTU1593 | 11778 | Low-quality | Unassigned | Unassigned | *Gammaproteobacteria* | Not determined |
| vOTU1595 | 16761 | Low-quality | Unassigned | Unassigned | *Gammaproteobacteria* | Not determined |
| vOTU1597 | 47599 | Medium-quality | Unassigned | Unassigned | *Chloroflexia* | Lysogeny |
| vOTU1598 | 11916 | Low-quality | Unassigned | Unassigned | *Gammaproteobacteria* | Not determined |
| vOTU1600 | 13567 | Low-quality | Unassigned | Unassigned | *Alphaproteobacteria* | Not determined |
| vOTU1601 | 24498 | Low-quality | Unassigned | Unassigned | *Gammaproteobacteria* | Not determined |
| vOTU1603 | 88059 | Medium-quality | Unassigned | Unassigned | *Gammaproteobacteria* | Lysogeny |
| vOTU1604 | 12263 | Low-quality | Unassigned | Unassigned | *Gammaproteobacteria* | Not determined |
| vOTU1605 | 33989 | Medium-quality | Unassigned | Unassigned | *Bacteroidia* | Not determined |
| vOTU1606 | 19868 | Low-quality | Unassigned | Unassigned | *Rhodothermia* | Not determined |
| vOTU1607 | 29628 | Low-quality | Unassigned | Unassigned | *Polyangia* | Not determined |
| vOTU1608 | 14094 | Low-quality | Unassigned | Unassigned | *Alphaproteobacteria* | Not determined |
| vOTU1609 | 36191 | Medium-quality | Unassigned | Unassigned | *Rhodothermia* | Not determined |
| vOTU1610 | 45050 | Medium-quality | Unassigned | Unassigned | *Gammaproteobacteria* | Lysogeny |
| vOTU1612 | 10292 | Low-quality | Unassigned | Unassigned | *Chloroflexia* | Not determined |
| vOTU1613 | 42402 | Medium-quality | Unassigned | Unassigned | *Nitrososphaeria* | Not determined |
| vOTU1614 | 11489 | Low-quality | Unassigned | Unassigned | *Gammaproteobacteria* | Not determined |
| vOTU1615 | 28004 | Low-quality | Unassigned | Unassigned | *Rhodothermia* | Not determined |
| vOTU1616 | 30185 | Medium-quality | Unassigned | Unassigned | *Gammaproteobacteria* | Not determined |
| vOTU1618 | 33631 | Low-quality | Unassigned | Unassigned | *Rhodothermia* | Not determined |
| vOTU1621 | 39824 | Medium-quality | Unassigned | Unassigned | *Actinomycetia* | Lysogeny |
| vOTU1623 | 10649 | Low-quality | Unassigned | Unassigned | *Chloroflexia* | Not determined |
| vOTU1627 | 38649 | Medium-quality | Unassigned | Unassigned | *Gammaproteobacteria* | Lysogeny |
| vOTU1628 | 11354 | Low-quality | Unassigned | Unassigned | *Alphaproteobacteria* | Not determined |
| vOTU1629 | 14777 | Low-quality | Unassigned | Unassigned | *Actinomycetia* | Not determined |
| vOTU1632 | 10478 | Low-quality | Unassigned | Unassigned | *Halobacteria* | Not determined |
| vOTU1633 | 11437 | Low-quality | Unassigned | Unassigned | *Rhodothermia* | Not determined |
| vOTU1634 | 55800 | Low-quality | Unassigned | Unassigned | *Rhodothermia* | Not determined |
| vOTU1635 | 36943 | Medium-quality | Unassigned | Unassigned | *Gammaproteobacteria* | Not determined |
| vOTU1638 | 14965 | Low-quality | Unassigned | Unassigned | *Gammaproteobacteria* | Not determined |
| vOTU1639 | 13855 | Low-quality | Unassigned | Unassigned | *Gammaproteobacteria* | Lysogeny |
| vOTU1640 | 10706 | Low-quality | Unassigned | Unassigned | *Rhodothermia* | Not determined |
| vOTU1641 | 27723 | Medium-quality | Caudovirales | Myoviridae | *Gammaproteobacteria* | Not determined |
| vOTU1643 | 37730 | Low-quality | Unassigned | Unassigned | *Rhodothermia* | Not determined |
| vOTU1644 | 25579 | Low-quality | Unassigned | Unassigned | *Gammaproteobacteria* | Lysogeny |
| vOTU1645 | 18071 | Low-quality | Unassigned | Unassigned | *Rhodothermia* | Not determined |
| vOTU1646 | 34223 | Low-quality | Unassigned | Unassigned | *Rhodothermia* | Not determined |
| vOTU1648 | 50410 | Medium-quality | Caudovirales | Myoviridae | *Gammaproteobacteria* | Not determined |
| vOTU1649 | 11506 | Low-quality | Unassigned | Unassigned | *Gammaproteobacteria* | Not determined |
| vOTU1651 | 11214 | Low-quality | Unassigned | Unassigned | *Alphaproteobacteria* | Not determined |
| vOTU1653 | 18348 | Low-quality | Unassigned | Unassigned | *Bacteroidia* | Not determined |
| vOTU1656 | 22192 | Low-quality | Unassigned | Unassigned | *Alphaproteobacteria* | Not determined |
| vOTU1657 | 10007 | Low-quality | Unassigned | Unassigned | *Gammaproteobacteria* | Not determined |
| vOTU1658 | 46082 | Medium-quality | Caudovirales | Myoviridae | *Gammaproteobacteria* | Not determined |
| vOTU1659 | 44946 | Medium-quality | Unassigned | Unassigned | *Gammaproteobacteria* | Not determined |
| vOTU1662 | 11205 | Low-quality | Unassigned | Unassigned | *Gammaproteobacteria* | Not determined |
| vOTU1663 | 21126 | Low-quality | Unassigned | Unassigned | *Gammaproteobacteria* | Not determined |
| vOTU1664 | 14293 | Low-quality | Unassigned | Unassigned | *Gammaproteobacteria* | Not determined |
| vOTU1665 | 17326 | Low-quality | Unassigned | Unassigned | *Gammaproteobacteria* | Not determined |
| vOTU1666 | 25204 | Low-quality | Unassigned | Unassigned | *SAR324* | Not determined |
| vOTU1667 | 13605 | Low-quality | Unassigned | Unassigned | *Rhodothermia* | Not determined |
| vOTU1668 | 49417 | Medium-quality | Unassigned | Unassigned | *Gammaproteobacteria* | Lysogeny |
| vOTU1671 | 58319 | Complete | Unassigned | Unassigned | *Gammaproteobacteria* | Lysogeny |
| vOTU1672 | 25360 | Low-quality | Unassigned | Unassigned | *Alphaproteobacteria* | Not determined |
| vOTU1673 | 36651 | Medium-quality | Unassigned | Unassigned | *Gammaproteobacteria* | Not determined |
| vOTU1675 | 14847 | Low-quality | Unassigned | Unassigned | *Gammaproteobacteria* | Not determined |
| vOTU1676 | 10204 | Low-quality | Unassigned | Unassigned | *Gammaproteobacteria* | Not determined |
| vOTU1678 | 28209 | Low-quality | Unassigned | Unassigned | *Gammaproteobacteria* | Not determined |
| vOTU1679 | 10390 | Low-quality | Unassigned | Unassigned | *Bacteroidia* | Not determined |
| vOTU1680 | 28884 | Low-quality | Unassigned | Unassigned | *Gammaproteobacteria* | Not determined |
| vOTU1681 | 39008 | Medium-quality | Unassigned | Unassigned | *Rhodothermia* | Not determined |
| vOTU1682 | 46056 | Medium-quality | Unassigned | Unassigned | *Bacteroidia* | Not determined |
| vOTU1683 | 39438 | Medium-quality | Unassigned | Unassigned | *Gammaproteobacteria* | Not determined |
| vOTU1684 | 49113 | Complete | Unassigned | Unassigned | *Gammaproteobacteria* | Not determined |
| vOTU1685 | 70190 | Medium-quality | Unassigned | Unassigned | *Halobacteria* | Not determined |
| vOTU1687 | 19210 | Low-quality | Unassigned | Unassigned | *SAR324* | Not determined |
| vOTU1691 | 10784 | Low-quality | Unassigned | Unassigned | *Gammaproteobacteria* | Not determined |
| vOTU1692 | 15506 | Low-quality | Unassigned | Unassigned | *Gammaproteobacteria* | Not determined |
| vOTU1693 | 31884 | Low-quality | Unassigned | Unassigned | *Gammaproteobacteria* | Not determined |
| vOTU1695 | 50058 | Medium-quality | Unassigned | Unassigned | *Actinomycetia* | Lysogeny |
| vOTU1696 | 16403 | Low-quality | Unassigned | Unassigned | *Gammaproteobacteria* | Not determined |
| vOTU1697 | 42531 | Medium-quality | Unassigned | Unassigned | *Bacilli* | Lysogeny |
| vOTU1700 | 23723 | Low-quality | Unassigned | Unassigned | *Actinomycetia* | Not determined |
| vOTU1701 | 33262 | Medium-quality | Unassigned | Unassigned | *Gammaproteobacteria* | Not determined |
| vOTU1702 | 16691 | Low-quality | Unassigned | Unassigned | *Gammaproteobacteria* | Not determined |
| vOTU1704 | 11176 | Low-quality | Unassigned | Unassigned | *Polyangia* | Not determined |
| vOTU1705 | 21547 | Low-quality | Unassigned | Unassigned | *Actinomycetia* | Not determined |
| vOTU1706 | 20580 | Low-quality | Unassigned | Unassigned | *Gammaproteobacteria* | Not determined |
| vOTU1707 | 24957 | Low-quality | Unassigned | Unassigned | *Actinomycetia* | Not determined |
| vOTU1708 | 47705 | Low-quality | Unassigned | Unassigned | *Gammaproteobacteria* | Not determined |
| vOTU1709 | 17342 | Low-quality | Unassigned | Unassigned | *Chloroflexia* | Not determined |
| vOTU1710 | 21323 | Low-quality | Unassigned | Unassigned | *Polyangia* | Not determined |
| vOTU1711 | 34504 | Low-quality | Unassigned | Unassigned | *Actinomycetia* | Not determined |
| vOTU1714 | 61072 | Medium-quality | Caudovirales | Podoviridae | *Gammaproteobacteria* | Not determined |
| vOTU1716 | 26694 | Low-quality | Unassigned | Unassigned | *Gammaproteobacteria* | Lysogeny |
| vOTU1717 | 40484 | Medium-quality | Unassigned | Unassigned | *Actinomycetia* | Lysogeny |
| vOTU1718 | 19383 | Low-quality | Unassigned | Unassigned | *Gammaproteobacteria* | Not determined |
| vOTU1719 | 24064 | Low-quality | Unassigned | Unassigned | *Gammaproteobacteria* | Not determined |
| vOTU1720 | 39799 | Low-quality | Unassigned | Unassigned | *Actinomycetia* | Lysogeny |
| vOTU1722 | 11802 | Low-quality | Unassigned | Unassigned | *Gammaproteobacteria* | Not determined |
| vOTU1724 | 14671 | Low-quality | Unassigned | Unassigned | *Gammaproteobacteria* | Not determined |
| vOTU1726 | 14924 | Low-quality | Unassigned | Unassigned | *Gammaproteobacteria* | Not determined |
| vOTU1727 | 26560 | Medium-quality | Unassigned | Unassigned | *Bacilli* | Not determined |
| vOTU1728 | 15012 | Low-quality | Unassigned | Unassigned | *Gammaproteobacteria* | Not determined |
| vOTU1729 | 41234 | Medium-quality | Unassigned | Unassigned | *Gammaproteobacteria* | Not determined |
| vOTU1733 | 11432 | Low-quality | Unassigned | Unassigned | *Gammaproteobacteria* | Not determined |
| vOTU1735 | 32555 | Medium-quality | Unassigned | Unassigned | *Gammaproteobacteria* | Not determined |
| vOTU1736 | 35408 | Medium-quality | Unassigned | Unassigned | *Gammaproteobacteria* | Not determined |
| vOTU1737 | 30750 | Low-quality | Caudovirales | Myoviridae | *Gammaproteobacteria* | Not determined |
| vOTU1738 | 22265 | Low-quality | Caudovirales | Myoviridae | *Gammaproteobacteria* | Not determined |
| vOTU1740 | 15944 | Low-quality | Unassigned | Unassigned | *Bacilli* | Not determined |
| vOTU1741 | 12625 | Low-quality | Unassigned | Unassigned | *Gammaproteobacteria* | Not determined |
| vOTU1742 | 14121 | Low-quality | Unassigned | Unassigned | *Gammaproteobacteria* | Not determined |
| vOTU1743 | 13583 | Low-quality | Unassigned | Unassigned | *Chloroflexia* | Not determined |
| vOTU1746 | 102109 | Medium-quality | Caudovirales | Siphoviridae | *Bacilli* | Lysogeny |
| vOTU1749 | 11065 | Low-quality | Unassigned | Unassigned | *Actinomycetia* | Not determined |
| vOTU1751 | 25917 | Low-quality | Unassigned | Unassigned | *Gammaproteobacteria* | Lysogeny |
| vOTU1752 | 25259 | Medium-quality | Unassigned | Unassigned | *Gammaproteobacteria* | Lysogeny |
| vOTU1754 | 89472 | Complete | Unassigned | Unassigned | *Actinomycetia* | Lysogeny |
| vOTU1755 | 41919 | Medium-quality | Caudovirales | Myoviridae | *Gammaproteobacteria* | Not determined |
| vOTU1756 | 54983 | Medium-quality | Unassigned | Unassigned | *Gammaproteobacteria* | Lysogeny |
| vOTU1757 | 10965 | Low-quality | Unassigned | Unassigned | *Actinomycetia* | Not determined |
| vOTU1758 | 41534 | Medium-quality | Caudovirales | Siphoviridae | *Gammaproteobacteria* | Not determined |
| vOTU1761 | 11307 | Low-quality | Unassigned | Unassigned | *Gammaproteobacteria* | Not determined |
| vOTU1762 | 23793 | Low-quality | Unassigned | Unassigned | *Gammaproteobacteria* | Not determined |
| vOTU1763 | 10616 | Low-quality | Unassigned | Unassigned | *Gammaproteobacteria* | Not determined |
| vOTU1765 | 16059 | Low-quality | Unassigned | Unassigned | *Gammaproteobacteria* | Not determined |
| vOTU1766 | 12591 | Low-quality | Unassigned | Unassigned | *Gammaproteobacteria* | Not determined |
| vOTU1767 | 15374 | Low-quality | Unassigned | Unassigned | *Actinomycetia* | Not determined |
| vOTU1769 | 30155 | Medium-quality | Unassigned | Unassigned | *Bacilli* | Not determined |
| vOTU1771 | 27655 | Low-quality | Unassigned | Unassigned | *Gammaproteobacteria* | Not determined |
| vOTU1774 | 10484 | Low-quality | Unassigned | Unassigned | *Gammaproteobacteria* | Not determined |
| vOTU1775 | 19597 | Low-quality | Unassigned | Unassigned | *Bacilli* | Not determined |
| vOTU1776 | 18330 | Low-quality | Unassigned | Unassigned | *Actinomycetia* | Not determined |
| vOTU1777 | 42509 | Medium-quality | Unassigned | Unassigned | *Actinomycetia* | Lysogeny |
| vOTU1779 | 21417 | Low-quality | Unassigned | Unassigned | *Gammaproteobacteria* | Not determined |
| vOTU1782 | 38130 | Medium-quality | Unassigned | Unassigned | *Gammaproteobacteria* | Not determined |
| vOTU1783 | 85552 | Medium-quality | Unassigned | Unassigned | *Rhodothermia* | Not determined |
| vOTU1784 | 39080 | Medium-quality | Unassigned | Unassigned | *Gammaproteobacteria* | Not determined |
| vOTU1785 | 15956 | Low-quality | Unassigned | Unassigned | *Gammaproteobacteria* | Not determined |
| vOTU1786 | 42589 | Medium-quality | Unassigned | Unassigned | *Actinomycetia* | Lysogeny |
| vOTU1787 | 24549 | Medium-quality | Caudovirales | Myoviridae | *Gammaproteobacteria* | Not determined |
| vOTU1789 | 38974 | Low-quality | Unassigned | Unassigned | *Actinomycetia* | Lysogeny |
| vOTU1790 | 39755 | Medium-quality | Unassigned | Unassigned | *Gammaproteobacteria* | Not determined |
| vOTU1794 | 58419 | Complete | Unassigned | Unassigned | *Gammaproteobacteria* | Lysogeny |
| vOTU1795 | 43498 | Complete | Unassigned | Unassigned | *Bacilli* | Lysogeny |
| vOTU1796 | 23490 | Low-quality | Unassigned | Unassigned | *Bacilli* | Not determined |
| vOTU1798 | 11745 | Low-quality | Unassigned | Unassigned | *Gammaproteobacteria* | Not determined |
| vOTU1801 | 11384 | Low-quality | Caudovirales | Myoviridae | *Gammaproteobacteria* | Not determined |
| vOTU1802 | 17076 | Low-quality | Unassigned | Unassigned | *Bacilli* | Not determined |
| vOTU1804 | 14688 | Low-quality | Unassigned | Unassigned | *Bacilli* | Not determined |
| vOTU1805 | 48942 | Medium-quality | Unassigned | Unassigned | *Gammaproteobacteria* | Not determined |
| vOTU1809 | 14022 | Low-quality | Caudovirales | Podoviridae | *Chloroflexia* | Not determined |
| vOTU1810 | 10181 | Low-quality | Unassigned | Unassigned | *Gammaproteobacteria* | Not determined |
| vOTU1811 | 28867 | Low-quality | Unassigned | Unassigned | *Actinomycetia* | Lysogeny |
| vOTU1812 | 57807 | Low-quality | Unassigned | Unassigned | *Bacilli* | Lysogeny |
| vOTU1813 | 15182 | Low-quality | Unassigned | Unassigned | *Gammaproteobacteria* | Not determined |
| vOTU1814 | 41972 | Medium-quality | Caudovirales | Myoviridae | *Gammaproteobacteria* | Not determined |
| vOTU1816 | 38863 | Low-quality | Unassigned | Unassigned | *Rhodothermia* | Not determined |
| vOTU1818 | 18047 | Low-quality | Unassigned | Unassigned | *Gammaproteobacteria* | Not determined |
| vOTU1823 | 29313 | Low-quality | Unassigned | Unassigned | *Alphaproteobacteria* | Lysogeny |
| vOTU1825 | 38209 | Low-quality | Caudovirales | Siphoviridae | *Bacilli* | Lysogeny |
| vOTU1826 | 46221 | Medium-quality | Unassigned | Unassigned | *Actinomycetia* | Lysogeny |
| vOTU1827 | 14420 | Low-quality | Unassigned | Unassigned | *Gammaproteobacteria* | Not determined |
| vOTU1828 | 13869 | Low-quality | Unassigned | Unassigned | *Bacteroidia* | Not determined |
| vOTU1829 | 11814 | Low-quality | Unassigned | Unassigned | *Bacteroidia* | Not determined |
| vOTU1831 | 21222 | Low-quality | Unassigned | Unassigned | *Actinomycetia* | Not determined |
| vOTU1832 | 10263 | Low-quality | Unassigned | Unassigned | *Alphaproteobacteria* | Not determined |
| vOTU1833 | 33539 | Medium-quality | Unassigned | Unassigned | *Rhodothermia* | Not determined |
| vOTU1834 | 19221 | Low-quality | Unassigned | Unassigned | *Bacilli* | Not determined |
| vOTU1835 | 20868 | Low-quality | Unassigned | Unassigned | *Gammaproteobacteria* | Not determined |
| vOTU1836 | 51574 | Medium-quality | Unassigned | Unassigned | *Bacteroidia* | Not determined |
| vOTU1838 | 11952 | Low-quality | Unassigned | Unassigned | *Bacilli* | Not determined |
| vOTU1839 | 19084 | Low-quality | Unassigned | Unassigned | *Actinomycetia* | Not determined |
| vOTU1840 | 14483 | Low-quality | Unassigned | Unassigned | *Bacilli* | Not determined |
| vOTU1841 | 13017 | Low-quality | Unassigned | Unassigned | *Bacilli* | Not determined |
| vOTU1842 | 19059 | Low-quality | Unassigned | Unassigned | *Gammaproteobacteria* | Not determined |
| vOTU1843 | 10437 | Low-quality | Unassigned | Unassigned | *Actinomycetia* | Not determined |
| vOTU1844 | 14964 | Not-determined | Unassigned | Unassigned | *Gammaproteobacteria* | Not determined |
| vOTU1845 | 12593 | Low-quality | Unassigned | Unassigned | *Gemmatimonadetes* | Not determined |
| vOTU1846 | 20235 | Low-quality | Unassigned | Unassigned | *Gammaproteobacteria* | Lysogeny |
| vOTU1847 | 10754 | Low-quality | Unassigned | Unassigned | *Rhodothermia* | Not determined |
| vOTU1849 | 18004 | Low-quality | Unassigned | Unassigned | *Bacilli* | Not determined |
| vOTU1850 | 30815 | Low-quality | Unassigned | Unassigned | *Gammaproteobacteria* | Lysogeny |
| vOTU1851 | 19646 | Low-quality | Caudovirales | Myoviridae | *Rhodothermia* | Not determined |
| vOTU1852 | 15733 | Low-quality | Unassigned | Unassigned | *Actinomycetia* | Not determined |
| vOTU1853 | 24955 | Low-quality | Unassigned | Unassigned | *Actinomycetia* | Not determined |
| vOTU1854 | 28296 | Low-quality | Unassigned | Unassigned | *Gammaproteobacteria* | Not determined |
| vOTU1856 | 12540 | Low-quality | Unassigned | Unassigned | *Bacilli* | Not determined |
| vOTU1857 | 14575 | Low-quality | Caudovirales | Siphoviridae | *Rhodothermia* | Not determined |
| vOTU1858 | 12840 | Low-quality | Unassigned | Unassigned | *Halobacteria* | Not determined |
| vOTU1859 | 66522 | Complete | Caudovirales | Siphoviridae | *Actinomycetia* | Not determined |
| vOTU1860 | 40488 | Complete | Caudovirales | Podoviridae | *Alphaproteobacteria* | Not determined |
| vOTU1861 | 61200 | Medium-quality | Caudovirales | Siphoviridae | *Actinomycetia* | Lysogeny |
| vOTU1862 | 37331 | Medium-quality | Unassigned | Unassigned | *Actinomycetia* | Not determined |
| vOTU1863 | 43438 | Medium-quality | Unassigned | Unassigned | *Gammaproteobacteria* | Lysogeny |
| vOTU1864 | 121821 | Medium-quality | Unassigned | Unassigned | *Bacilli* | Not determined |
| vOTU1869 | 51853 | Medium-quality | Unassigned | Unassigned | *Bacteroidia* | Not determined |
| vOTU1870 | 13245 | Low-quality | Unassigned | Unassigned | *Actinomycetia* | Not determined |
| vOTU1871 | 75392 | Medium-quality | Unassigned | Unassigned | *Actinomycetia* | Lysogeny |
| vOTU1872 | 81518 | Complete | Unassigned | Unassigned | *Chloroflexia* | Not determined |
| vOTU1873 | 42086 | Medium-quality | Unassigned | Unassigned | *Gemmatimonadetes* | Not determined |
| vOTU1875 | 59333 | Medium-quality | Unassigned | Unassigned | *Gammaproteobacteria* | Lysogeny |
| vOTU1877 | 29047 | Low-quality | Unassigned | Unassigned | *Gammaproteobacteria* | Not determined |
| vOTU1878 | 28298 | Low-quality | Caudovirales | Siphoviridae | *Bacilli* | Not determined |
| vOTU1880 | 13322 | Low-quality | Unassigned | Unassigned | *Rhodothermia* | Not determined |
| vOTU1882 | 26662 | Low-quality | Unassigned | Unassigned | *Gammaproteobacteria* | Lysogeny |
| vOTU1884 | 29186 | Medium-quality | Caudovirales | Myoviridae | *Gammaproteobacteria* | Not determined |
| vOTU1886 | 16355 | Low-quality | Unassigned | Unassigned | *Gammaproteobacteria* | Not determined |
| vOTU1887 | 29677 | Low-quality | Unassigned | Unassigned | *Polyangia* | Not determined |
| vOTU1888 | 74453 | Medium-quality | Unassigned | Unassigned | *Actinomycetia* | Lysogeny |
| vOTU1889 | 18634 | Low-quality | Unassigned | Unassigned | *Gammaproteobacteria* | Not determined |
| vOTU1890 | 30562 | Low-quality | Unassigned | Unassigned | *Gammaproteobacteria* | Lysogeny |
| vOTU1892 | 12638 | Low-quality | Unassigned | Unassigned | *Gammaproteobacteria* | Not determined |
| vOTU1893 | 19632 | Low-quality | Unassigned | Unassigned | *Actinomycetia* | Not determined |
| vOTU1894 | 160047 | Medium-quality | Unassigned | Unassigned | *Rhodothermia* | Lysogeny |
| vOTU1896 | 46957 | Medium-quality | Unassigned | Unassigned | *Gammaproteobacteria* | Not determined |
| vOTU1897 | 19368 | Low-quality | Unassigned | Unassigned | *Bacteroidia* | Not determined |
| vOTU1898 | 14791 | Low-quality | Unassigned | Unassigned | *Gammaproteobacteria* | Not determined |
| vOTU1899 | 57548 | Medium-quality | Unassigned | Unassigned | *Actinomycetia* | Not determined |
| vOTU1900 | 53426 | Medium-quality | Unassigned | Unassigned | *Actinomycetia* | Not determined |
| vOTU1901 | 10981 | Low-quality | Unassigned | Unassigned | *Gemmatimonadetes* | Not determined |
| vOTU1902 | 51914 | Medium-quality | Caudovirales | Podoviridae | *Bacteroidia* | Not determined |
| vOTU1904 | 21233 | Low-quality | Unassigned | Unassigned | *Actinomycetia* | Not determined |
| vOTU1906 | 20893 | Low-quality | Unassigned | Unassigned | *Actinomycetia* | Not determined |
| vOTU1907 | 25928 | Low-quality | Unassigned | Unassigned | *Gammaproteobacteria* | Not determined |
| vOTU1909 | 11309 | Not-determined | Unassigned | Unassigned | *Rhodothermia* | Not determined |
| vOTU1910 | 35950 | Low-quality | Unassigned | Unassigned | *Actinomycetia* | Not determined |
| vOTU1911 | 38512 | Medium-quality | Unassigned | Unassigned | *Gammaproteobacteria* | Lysogeny |
| vOTU1912 | 12549 | Low-quality | Unassigned | Unassigned | *Bacteroidia* | Not determined |
| vOTU1913 | 68954 | Low-quality | Unassigned | Unassigned | *Rhodothermia* | Not determined |
| vOTU1914 | 22045 | Low-quality | Unassigned | Unassigned | *Rhodothermia* | Not determined |
| vOTU1915 | 19322 | Low-quality | Unassigned | Unassigned | *Actinomycetia* | Not determined |
| vOTU1916 | 38177 | Medium-quality | Unassigned | Unassigned | *Gammaproteobacteria* | Lysogeny |
| vOTU1918 | 38455 | Medium-quality | Unassigned | Unassigned | *Rhodothermia* | Not determined |
| vOTU1919 | 40457 | Low-quality | Unassigned | Unassigned | *Gammaproteobacteria* | Not determined |
| vOTU1920 | 39652 | Medium-quality | Unassigned | Unassigned | *Rhodothermia* | Not determined |
| vOTU1921 | 22686 | Low-quality | Unassigned | Unassigned | *Bacilli* | Not determined |
| vOTU1922 | 12409 | Low-quality | Caudovirales | Siphoviridae | *Actinomycetia* | Not determined |
| vOTU1924 | 28105 | Low-quality | Unassigned | Unassigned | *Gammaproteobacteria* | Not determined |
| vOTU1926 | 28515 | Low-quality | Unassigned | Unassigned | *Actinomycetia* | Not determined |
| vOTU1927 | 35928 | Medium-quality | Unassigned | Unassigned | *Actinomycetia* | Not determined |
| vOTU1928 | 32275 | Low-quality | Unassigned | Unassigned | *Gammaproteobacteria* | Not determined |
| vOTU1929 | 17334 | Low-quality | Unassigned | Unassigned | *Actinomycetia* | Not determined |
| vOTU1930 | 11173 | Low-quality | Unassigned | Unassigned | *Gammaproteobacteria* | Not determined |
| vOTU1931 | 10182 | Low-quality | Unassigned | Unassigned | *Bacilli* | Not determined |
| vOTU1932 | 68405 | Low-quality | Unassigned | Unassigned | *Rhodothermia* | Not determined |
| vOTU1933 | 46195 | Medium-quality | Unassigned | Unassigned | *Gammaproteobacteria* | Lysogeny |
| vOTU1935 | 38599 | Low-quality | Unassigned | Unassigned | *Bacteroidia* | Not determined |
| vOTU1936 | 162193 | High-quality | Unassigned | Unassigned | *Gammaproteobacteria* | Not determined |
| vOTU1937 | 34452 | Medium-quality | Unassigned | Unassigned | *Gemmatimonadetes* | Not determined |
| vOTU1938 | 12077 | Low-quality | Unassigned | Unassigned | *Chloroflexia* | Not determined |
| vOTU1939 | 10596 | Low-quality | Unassigned | Unassigned | *Chloroflexia* | Not determined |
| vOTU1940 | 21585 | Low-quality | Unassigned | Unassigned | *Actinomycetia* | Not determined |
| vOTU1941 | 46696 | Medium-quality | Caudovirales | Siphoviridae | *Gemmatimonadetes* | Lysogeny |
| vOTU1942 | 51326 | Medium-quality | Unassigned | Unassigned | *Thermoanaerobaculia* | Not determined |
| vOTU1945 | 10418 | Low-quality | Unassigned | Unassigned | *Bacilli* | Not determined |
| vOTU1947 | 10044 | Low-quality | Unassigned | Unassigned | *Bacteroidia* | Not determined |
| vOTU1948 | 17704 | Medium-quality | Unassigned | Unassigned | *Rhodothermia* | Not determined |
| vOTU1949 | 14525 | Low-quality | Unassigned | Unassigned | *Chloroflexia* | Not determined |
| vOTU1951 | 51468 | Medium-quality | Unassigned | Unassigned | *Gemmatimonadetes* | Not determined |
| vOTU1952 | 16446 | Low-quality | Unassigned | Unassigned | *Gammaproteobacteria* | Lysogeny |
| vOTU1953 | 60387 | Low-quality | Unassigned | Unassigned | *Gammaproteobacteria* | Not determined |
