# Supplementary figures and images for: Viral Community Structure and Potential Functions in the Dried-Out Aral Sea Basin Change along a Desiccation Gradient
Source: mSystems. 2023 Jan 10;8(1):e00994-22. doi: 10.1128/msystems.00994-22 (PMC9948696; doi:10.1128/msystems.00994-22)

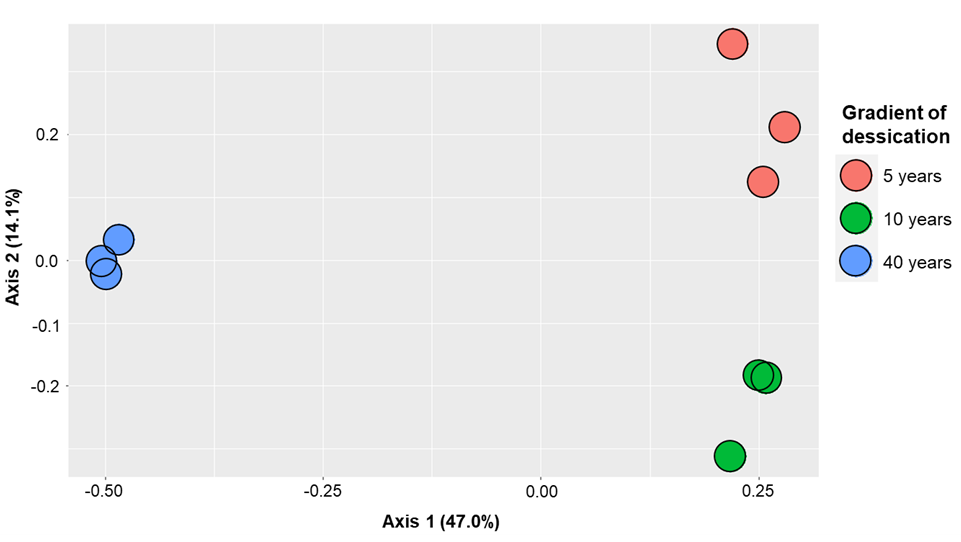

Supplement: FIG S1 [file msystems.00994-22-s0002.tif]

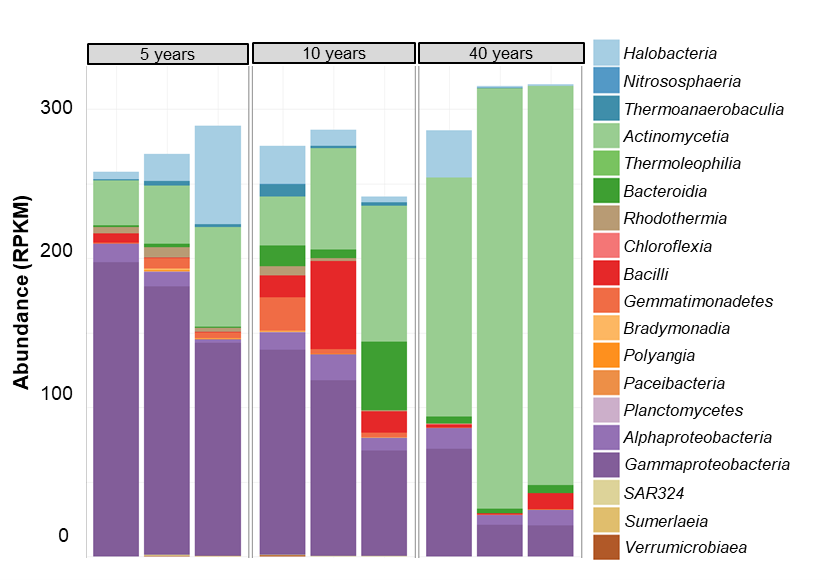

Supplement: FIG S2 [file msystems.00994-22-s0003.tif]
