# Supplementary material for: Viral Community Structure and Potential Functions in the Dried-Out Aral Sea Basin Change along a Desiccation Gradient
Source: mSystems. 2023 Jan 10;8(1):e00994-22. doi: 10.1128/msystems.00994-22 (PMC9948696; doi:10.1128/msystems.00994-22)
Supplement: TABLE S2 [file msystems.00994-22-s0005.docx]

**Supplementary Table S2. Details of metagenome assembled genomes (MAGs)**

| MAG ID | Completeness (contamination) % | Taxonomical identification | | Abundance (RPKM) | | |
| --- | --- | --- | --- | --- | --- | --- |
|  |  | Genus | Class | 5 years | 10 years | 40 years |
| MAG1 | 85.24 (8.38) | *Arthrobacter_D* | *Actinomycetia* | 0.02 | 0.09 | 7.82 |
| MAG2 | 99.73 (0.05) | *Saccharibacillus* | *Bacilli* | 0.03 | 0.07 | 4.17 |
| MAG3 | 83.09 (6.86) | *Pontixanthobacter* | *Alphaproteobacteria* | 2.23 | 1.93 | 4.72 |
| MAG4 | 96.3 (5.38) | *Nitratireductor_A* | *Alphaproteobacteria* | 0.08 | 0.12 | 2.83 |
| MAG5 | 99.57 (2.37) | *Halomonas* | *Gammaproteobacteria* | 4.74 | 1.90 | 14.96 |
| MAG6 | 94.1 (3.31) | *Halomonas* | *Gammaproteobacteria* | 55.18 | 33.94 | 5.76 |
| MAG7 | 95.3 (7.03) | *Halopelagius* | *Halobacteria* | 0.23 | 0.11 | 0.89 |
| MAG8 | 99.64 (0.95) | *Halomonas* | *Gammaproteobacteria* | 10.74 | 6.48 | 1.44 |
| MAG9 | 87.91 (1.53) | *Nocardiopsis* | *Actinomycetia* | 0.26 | 2.46 | 0.60 |
| MAG10 | 95.33 (2.16) | *Halomonas* | *Gammaproteobacteria* | 13.07 | 6.42 | 0.80 |
| MAG11 | 88.89 (7.3) | *Paracoccus* | *Alphaproteobacteria* | 0.12 | 0.84 | 0.40 |
| MAG12 | 98.99 (2.62) | *Sinomicrobium* | *Bacteroidia* | 0.13 | 14.65 | 0.21 |
| MAG13 | 91.1 (2.17) | *Microbulbifer* | *Gammaproteobacteria* | 7.89 | 5.48 | 2.75 |
| MAG14 | 98.84 (1.98) | *Sinomicrobium* | *Bacteroidia* | 0.11 | 6.98 | 0.10 |
| MAG15 | 98.58 (0.8) | *Amycolatopsis_C* | *Actinomycetia* | 2.24 | 4.29 | 0.42 |
| MAG16 | 78.45 (6.9) | *Zhihengliuella* | *Actinomycetia* | 1.27 | 12.99 | 2.65 |
| MAG17 | 95.56 (1.45) | *Nitratireductor* | *Alphaproteobacteria* | 0.23 | 1.08 | 0.51 |
| MAG18 | 83.03 (3.19) | *Unc. UBA6522* | *Gammaproteobacteria* | 0.32 | 1.63 | 0.02 |
| MAG19 | 97.83 (1.13) | *Nesterenkonia* | *Actinomycetia* | 6.68 | 2.57 | 72.96 |
| MAG20 | 97.44 (0.85) | *Unc. UBA5704* | *Thermoanaerobaculia* | 1.11 | 1.96 | 0.06 |
| MAG21 | 93.23 (1.71) | *Paracoccus* | *Alphaproteobacteria* | 0.08 | 1.57 | 0.23 |
| MAG22 | 87.99 (8.25) | *Unc. Haladaptataceae* | *Halobacteria* | 0.54 | 1.38 | 0.03 |
| MAG23 | 99.42 (5.5) | *Halomonas* | *Gammaproteobacteria* | 46.27 | 1.94 | 0.18 |
| MAG24 | 92.13 (2.59) | *Natronoarchaeum* | *Halobacteria* | 7.31 | 1.52 | 0.32 |
| MAG25 | 90.9 (3.82) | *Marinimicrobium* | *Gammaproteobacteria* | 2.95 | 2.00 | 0.26 |
| MAG26 | 97.71 (2.26) | *Amycolatopsis_C* | *Actinomycetia* | 2.82 | 0.16 | 1.25 |
| MAG27 | 83.54 (1.69) | *BRH-c0* | *Gammaproteobacteria* | 1.59 | 0.26 | 0.03 |
| MAG28 | 98.09 (1.22) | *Sediminimonas* | *Alphaproteobacteria* | 1.48 | 1.34 | 0.06 |
| MAG29 | 89.66 (1.35) | *Algiphilus* | *Gammaproteobacteria* | 0.99 | 0.24 | 0.04 |
| MAG30 | 86.87 (3.83) | *Unc. Balneolaceae* | *Rhodothermia* | 3.87 | 0.65 | 0.11 |
| MAG31 | 99.44 (0.83) | *Staphylococcus* | *Bacilli* | 2.03 | 3.38 | 0.17 |
| MAG32 | 96.63 (2.22) | *Pseudidiomarina* | *Gammaproteobacteria* | 0.95 | 0.31 | 0.00 |
| MAG33 | 95.53 (4.09) | *Parahaliea* | *Gammaproteobacteria* | 1.99 | 0.83 | 0.06 |
| MAG34 | 90.71 (3.07) | *BRH-c0* | *Gammaproteobacteria* | 1.69 | 0.27 | 0.02 |
| MAG35 | 77.49 (3.83) | *Halobacterium* | *Halobacteria* | 3.32 | 0.42 | 0.15 |
| MAG36 | 89.94 (5.61) | *Nesterenkonia* | *Actinomycetia* | 13.29 | 0.27 | 12.81 |
| MAG37 | 81.8 (3.64) | *Unc. Xanthomonadales* | *Gammaproteobacteria* | 1.76 | 4.56 | 0.47 |
| MAG38 | 84.25 (2.97) | *Unc. Pseudonocardiaceae* | *Actinomycetia* | 1.45 | 0.97 | 1.34 |
| MAG39 | 92.58 (5.23) | *Salegentibacter* | *Bacteroidia* | 0.65 | 0.08 | 0.03 |
| MAG40 | 78.95 (1.09) | *Pontixanthobacter* | *Alphaproteobacteria* | 0.64 | 0.29 | 0.56 |
| MAG41 | 91.3 (2.38) | *Nocardiopsis_A* | *Actinomycetia* | 4.15 | 0.48 | 0.41 |
| MAG42 | 91.57 (2.36) | *Chelativorans* | *Alphaproteobacteria* | 1.57 | 3.06 | 0.15 |
| MAG43 | 97.65 (1.18) | *Unc. Jiangellaceae* | *Actinomycetia* | 1.39 | 0.71 | 0.15 |
| MAG44 | 86.13 (4.04) | *Halobaculum* | *Halobacteria* | 2.23 | 0.45 | 0.28 |
| MAG45 | 86.29 (4.6) | *Haloarcula* | *Halobacteria* | 11.46 | 0.37 | 0.13 |
| MAG46 | 95.6 (3.3) | *Unc. KS3-K002* | *Gemmatimonadetes* | 1.68 | 3.96 | 0.08 |
| MAG47 | 97.47 (6.19) | *Cytobacillus* | *Bacilli* | 0.06 | 1.38 | 0.11 |
| MAG48 | 88.61 (2.88) | *Unc. UBA10348* | *Rhodothermia* | 0.26 | 0.90 | 0.00 |
| MAG49 | 84.05 (7.17) | *Unc. Haladaptataceae* | *Halobacteria* | 0.51 | 1.20 | 0.05 |
| MAG50 | 85.89 (7.45) | *Unc. GCA-2696645* | *Alphaproteobacteria* | 1.23 | 1.35 | 0.09 |
| MAG51 | 90.85 (0.97) | *Unc. UBA4486* | *Gammaproteobacteria* | 0.40 | 2.21 | 0.12 |
| MAG52 | 76.95 (3.42) | *Unc. Nitriliruptorales* | *Actinomycetia* | 1.14 | 2.53 | 0.29 |
| MAG53 | 97.89 (2.85) | *Halobacterium* | *Halobacteria* | 1.80 | 4.60 | 0.14 |
| MAG54 | 95.6 (3.84) | *Nocardioides* | *Actinomycetia* | 0.03 | 8.55 | 0.81 |
| MAG55 | 79.89 (8.88) | *Halofilum* | *Gammaproteobacteria* | 4.76 | 4.28 | 0.08 |
| MAG56 | 94.38 (0.56) | *Marinimicrobium* | *Gammaproteobacteria* | 0.27 | 3.31 | 0.10 |
| MAG57 | 89.46 (9.5) | *Unc. UBA6960* | *Gemmatimonadetes* | 0.24 | 1.07 | 0.01 |
| MAG58 | 99.14 (4.56) | *Staphylococcus* | *Bacilli* | 0.04 | 24.60 | 0.10 |
| MAG59 | 96.84 (1.36) | *Glycomyces* | *Actinomycetia* | 0.46 | 3.06 | 2.57 |
| MAG60 | 85.36 (2.7) | *Myceligenerans* | *Actinomycetia* | 0.27 | 12.06 | 3.90 |
| MAG61 | 90.16 (2.08) | *Alcanivorax* | *Gammaproteobacteria* | 0.84 | 3.35 | 0.06 |
| MAG62 | 94.07 (3.74) | *Microbacterium* | *Actinomycetia* | 0.28 | 1.62 | 0.28 |
| MAG63 | 83.99 (3.3) | *Paracoccus* | *Alphaproteobacteria* | 0.14 | 0.47 | 0.75 |
| MAG64 | 92.05 (2.49) | *Microbacterium* | *Actinomycetia* | 0.04 | 0.85 | 0.83 |
| MAG65 | 90.32 (1.85) | *Saccharospirillum* | *Gammaproteobacteria* | 0.31 | 0.86 | 1.86 |
| MAG66 | 91.51 (4.06) | *Halopelagius* | *Halobacteria* | 0.25 | 0.11 | 1.02 |
| MAG67 | 89.83 (5.07) | *Arthrobacter_D* | *Actinomycetia* | 0.04 | 0.10 | 8.43 |
| MAG68 | 97.1 (3.32) | *Halomarina* | *Halobacteria* | 0.61 | 0.39 | 2.93 |
| MAG69 | 85.76 (4.24) | *Kocuria* | *Actinomycetia* | 8.11 | 8.14 | 118.96 |
| MAG70 | 85.79 (1.01) | *Paucimonas* | *Gammaproteobacteria* | 0.92 | 0.73 | 6.28 |
| MAG71 | 99.36 (1.91) | *Halalkalicoccus* | *Halobacteria* | 0.35 | 0.22 | 5.40 |
| MAG72 | 90.75 (2.28) | *NIC37A-2* | *Polyangia* | 0.43 | 0.22 | 0.01 |
| MAG73 | 82.95 (3.41) | *Unc. Halobacteriaceae* | *Halobacteria* | 0.74 | 2.41 | 0.09 |
| MAG74 | 90.17 (0.47) | *REBR01* | *Gammaproteobacteria* | 1.04 | 2.43 | 0.04 |
| MAG75 | 84.8 (1.88) | *Halomonas* | *Gammaproteobacteria* | 3.48 | 4.28 | 0.21 |
| MAG76 | 78.25 (4.99) | *Unc. SAR324* | *SAR324* | 0.26 | 0.23 | 0.01 |
| MAG77 | 88.42 (6.82) | *Persicimonas* | *Bradymonadia* | 0.45 | 0.27 | 0.00 |
| MAG78 | 83.23 (5.84) | *Unc. Xanthomonadales* | *Gammaproteobacteria* | 1.51 | 4.29 | 0.45 |
| MAG79 | 81.03 (5.93) | *Unc. Cyclobacteriaceae* | *Bacteroidia* | 0.08 | 0.06 | 0.00 |
| MAG80 | 75.5 (3.52) | *Unc. Pedosphaerales* | *Verrucomicrobiae* | 0.13 | 0.32 | 0.00 |
| MAG81 | 85.77 (5.56) | *Unc. Sumerlaeia* | *Sumerlaeia* | 0.08 | 0.07 | 0.00 |
| MAG82 | 95.29 (1.14) | *Methyloceanibacter* | *Alphaproteobacteria* | 0.30 | 0.34 | 0.03 |
| MAG83 | 82.84 (1.9) | *Unc. SLND01* | *Gammaproteobacteria* | 0.29 | 0.27 | 0.03 |
| MAG84 | 87.05 (6.08) | *Unc. Thiohalomonadales* | *Gammaproteobacteria* | 0.26 | 0.28 | 0.05 |
| MAG85 | 87.6 (4.47) | *Natronomonas* | *Halobacteria* | 0.33 | 0.26 | 0.01 |
| MAG86 | 97.8 (4.4) | *Unc. UBA6960* | *Gemmatimonadetes* | 0.84 | 0.83 | 0.01 |
| MAG87 | 91.41 (0.21) | *Unc. UBA1268* | *Planctomycetes* | 0.13 | 0.12 | 0.00 |
| MAG88 | 91.44 (6.44) | *Unc. HTCC2089* | *Gammaproteobacteria* | 0.16 | 0.07 | 0.01 |
| MAG89 | 76.93 (2.26) | *Unc. Arenicellales* | *Gammaproteobacteria* | 0.95 | 1.39 | 0.14 |
| MAG90 | 88.55 (8.52) | *Thiohalophilus* | *Gammaproteobacteria* | 0.43 | 0.36 | 0.02 |
| MAG91 | 87.15 (1.1) | *Unc. Sedimenticolaceae* | *Gammaproteobacteria* | 0.16 | 0.12 | 0.07 |
| MAG92 | 90.74 (1.78) | *Unc. UBA5704* | *Thermoanaerobaculia* | 0.66 | 1.85 | 0.04 |
| MAG93 | 77.03 (9.79) | *Salegentibacter* | *Bacteroidia* | 0.46 | 0.07 | 0.02 |
| MAG94 | 87.78 (7.63) | *UBA2589* | *Gemmatimonadetes* | 0.89 | 1.48 | 0.04 |
| MAG95 | 81.36 (3.27) | *Wenzhouxiangella* | *Gammaproteobacteria* | 0.86 | 0.25 | 0.01 |
| MAG96 | 90.29 (2.02) | *Unc. Nitrosopumilaceae* | *Nitrososphaeria* | 0.09 | 0.18 | 0.00 |
| MAG97 | 75.99 (3.45) | *Unc. UBA9983_A* | *Paceibacteria* | 0.01 | 0.02 | 0.00 |
| MAG98 | 92.92 (4.16) | *Unc. HTCC2089* | *Gammaproteobacteria* | 0.09 | 0.23 | 0.02 |
| MAG99 | 89.94 (1.46) | *Unc. Arenicellales* | *Gammaproteobacteria* | 1.71 | 7.53 | 0.42 |
| MAG100 | 88.25 (4.52) | *Unc. Balneolaceae* | *Rhodothermia* | 0.38 | 0.66 | 0.03 |
| MAG101 | 81.11 (3.56) | *Unc. Xanthomonadales* | *Gammaproteobacteria* | 0.22 | 0.79 | 0.57 |
| MAG102 | 91.55 (1.94) | *Salinibacter* | *Rhodothermia* | 0.05 | 0.42 | 0.00 |
| MAG103 | 83.83 (2.02) | *YR4-1* | *Rhodothermia* | 0.17 | 0.34 | 0.00 |
| MAG104 | 85.47 (2.14) | *Unc. Nitriliruptoraceae* | *Actinomycetia* | 1.38 | 2.08 | 0.20 |
| MAG105 | 92.26 (2.95) | *Halofilum* | *Gammaproteobacteria* | 4.62 | 5.38 | 0.08 |
| MAG106 | 89.56 (2.2) | *Unc. JABDQB01* | *Gemmatimonadetes* | 0.31 | 2.19 | 0.05 |
| MAG107 | 86.8 (2.89) | *Pontibacter* | *Bacteroidia* | 0.01 | 0.05 | 3.85 |
| MAG108 | 78.27 (3.04) | *Allosphingosinicella* | *Alphaproteobacteria* | 0.05 | 0.06 | 0.10 |
| MAG109 | 87.69 (3.5) | *Unc. Burkholderiaceae* | *Gammaproteobacteria* | 0.01 | 0.03 | 0.66 |
| MAG110 | 92.46 (4.29) | *Unc. Cyclobacteriaceae* | *Bacteroidia* | 0.01 | 0.00 | 0.06 |
| MAG111 | 92.46 (6.47) | *Unc. SKUG01* | *Thermoleophilia* | 0.00 | 0.01 | 0.04 |
| MAG112 | 95.28 (0.16) | *JACCVC01* | *Chloroflexia* | 0.00 | 0.00 | 0.17 |
