# Supplementary material for: Viral Community Structure and Potential Functions in the Dried-Out Aral Sea Basin Change along a Desiccation Gradient
Source: mSystems. 2023 Jan 10;8(1):e00994-22. doi: 10.1128/msystems.00994-22 (PMC9948696; doi:10.1128/msystems.00994-22)
Supplement: TABLE S3 [file msystems.00994-22-s0006.docx]

**Supplementary Table S3. Details of auxiliary metabolic genes (AMGs) from viral population (vOTUs)**

| vOTU ID | Host | Gene description | KEGG functional categories | KEGG functional categories |
| --- | --- | --- | --- | --- |
| 194 | Nitrososphaeria | nicotinamide phosphoribosyltransferase | Nicotinate and nicotinamide metab. | Metab. Of cofactors and vitamins |
| 194 | Nitrososphaeria | CBH2, cbhA; cellulose 1,4-beta-cellobiosidase | Starch and sucrose metab. | Carbohydrate metab. |
| 194 | Nitrososphaeria | DHFR, folA; dihydrofolate reductase | Folate biosynthesis | metab. Of cofactors and vitamins |
| 238 | Actinomycetia | Protein of unknown function (DUF2867) | - | - |
| 286 | Gammaproteobacteria | cysH; phosphoadenosine phosphosulfate reductase | Sulfur metab. | Energy metab. |
| 311 | Gammaproteobacteria | branched-chain amino acid transport system permease | ABC transporters | Membrane transport |
| 311 | Gammaproteobacteria | branched-chain amino acid transport system permease | ABC transporters | Membrane transport |
| 311 | Gammaproteobacteria | branched-chain amino acid transport system substrate-binding | ABC transporters | Membrane transport |
| 346 | Gammaproteobacteria | NitT/TauT family transport system substrate-binding protein | Transporters |  |
| 352 | Chloroflexia | cobaltochelatase CobS | Porphyrin metab. | Metab. Of cofactors and vitamins |
| 352 | Chloroflexia | cobaltochelatase CobT | Porphyrin metab. | Metab. Of cofactors and vitamins |
| 364 | Actinomycetia | DBT, bkdB | Propanoate metab. | Carbohydrate metab. |
| 371 | Polyangia | GTP cyclohydrolase IA | Folate biosynthesis | metab. Of cofactors and vitamins |
| 419 | Gammaproteobacteria | asnB, ASNS; asparagine synthase (glutamine-hydrolysing) | Alanine, aspartate and glutamate metab. | Amino acid metab. |
| 551 | Gammaproteobacteria | moeB; molybdopterin-synthase adenylyltransferase | Sulfur relay system | Folding, sorting and degradation |
| 723 | Gammaproteobacteria | cysteine desulfurase / selenocysteine lyase | Selenocompound metab. | Metab. Of other amino acids |
| 758 | Actinomycetia | cysH; phosphoadenosine phosphosulfate reductase | Sulfur metab. | Energy metab. |
| 761 | Alphaproteobacteria | Sulfotransferase family | - | - |
| 812 | Gammaproteobacteria | enolase | Glycolysis / Gluconeogenesis | Carbohydrate metab. |
| 828 | Actinomycetia | alkaline phosphatase D | Folate biosynthesis | metab. Of cofactors and vitamins |
| 860 | Gammaproteobacteria | 7-cyano-7-deazaguanine reductase | Folate biosynthesis | metab. Of cofactors and vitamins |
| 900 | Gammaproteobacteria | 8-amino-7-oxononanoate synthase | Biotin metab. | Metab. Of cofactors and vitamins |
| 1062 | Actinomycetia | pcaD; 3-oxoadipate enol-lactonase | Benzoate degradation | Xenobiotics biodegradation and metab. |
| 1112 | Bacilli | Cellulase GH5 | Glycosyl hydrolases | Carbohydrate metab. |
| 1112 | Bacilli | Cellulase GH5 | Glycosyl hydrolases | Carbohydrate metab. |
| 1135 | Halobacteria | cysH; phosphoadenosine phosphosulfate reductase | Sulfur metab. | Energy metab. |
| 1154 | Gammaproteobacteria | membrane fusion protein, hemolysin D | Bacterial secretion system | Membrane transport |
| 1156 | Bacilli | Cellulase GH5 | Glycosyl hydrolases | Carbohydrate metab. |
| 1199 | Gammaproteobacteria | membrane fusion protein, hemolysin D | Bacterial secretion system | Membrane transport |
| 1234 | Actinomycetia | GH5 endo-beta-1,4-glucanase / cellulase | Glycosyl hydrolases | Carbohydrate metab. |
| 1254 | Rhodothermia | serine O-acetyltransferase | Sulfur metab. | Energy metab. |
| 1353 | Actinomycetia | GH6 endoglucanase ; cellobiohydrolase | Glycosyl hydrolases | Carbohydrate metab. |
| 1353 | Actinomycetia | PL7 poly(beta-mannuronate) lyase | Fructose and mannose metab. | Carbohydrate metab. |
| 1388 | Actinomycetia | GH87 mycodextranase; alpha-1,3-glucanase | Glycosyl hydrolases | Carbohydrate metab. |
| 1388 | Actinomycetia | CE4 acetyl xylan esterase; chitin deacetylase | Amino sugar and nucleotide sugar metab. |  |
| 1454 | SAR324 | GTP cyclohydrolase IA | Folate biosynthesis | metab. Of cofactors and vitamins |
| 1454 | SAR324 | 6-pyruvoyltetrahydropterin/6-carboxytetrahydropterin synthase | Folate biosynthesis | metab. Of cofactors and vitamins |
| 1495 | Actinomycetia | GH26 beta-mannanase | Glycosyl hydrolases | Carbohydrate metab. |
| 1511 | Bacilli | phnP; phosphoribosyl 1,2-cyclic phosphate phosphodiesterase | Phosphonate and phosphinate metab. | Metab. Of other amino acids |
| 1511 | Bacilli | GH3 beta-glucosidase; xylan 1,4-beta-xylosidase | Glycosyl hydrolases | Carbohydrate metab. |
| 1525 | Actinomycetia | dcyD; D-cysteine desulfhydrase | Cysteine and methionine metab. | Amino acid metab. |
| 1574 | Actinomycetia | E4.2.1.46, rfbB, rffG | O-Antigen nucleotide sugar biosynthesis | Glycan biosynthesis and metab. |
| 1581 | Rhodothermia | waaL, rfaL; O-antigen ligase | Lipopolysaccharide biosynthesis | Glycan biosynthesis and metab. |
| 1628 | Alphaproteobacteria | gloB, gloC, HAGH; hydroxyacylglutathione hydrolase | Pyruvate metab. | Carbohydrate metab. |
| 1658 | Gammaproteobacteria | cysH; phosphoadenosine phosphosulfate reductase | Sulfur metab. | Energy metab. |
| 1673 | Gammaproteobacteria | Sulfotransferase family | - | - |
| 1673 | Gammaproteobacteria | rfbD, rmlD; dTDP-4-dehydrorhamnose reductase | O-Antigen nucleotide sugar biosynthesis | Glycan biosynthesis and metab. |
| 1684 | Gammaproteobacteria | alkaline phosphatase D | Folate biosynthesis | metab. Of cofactors and vitamins |
| 1709 | Chloroflexia | horismite mutase | Phenylalanine, tyrosine and tryptophan metab. | Amino acid metab. |
| 1754 | Actinomycetia | Aldo/keto reductase family | - | - |
| 1754 | Actinomycetia | quinolinate synthase | Nicotinate and nicotinamide metab. | Metab. Of cofactors and vitamins |
| 1754 | Actinomycetia | cysteine desulfurase | Selenocompound metab. | Metab. Of other amino acids |
| 1754 | Actinomycetia | cytochrome c oxidase subunit II | Oxidative phosphorylation | Energy metab. |
| 1754 | Actinomycetia | anthranilate phosphoribosyltransferase | Phenylalanine, tyrosine and tryptophan metab. | Amino acid metabolism |
| 1756 | Gammaproteobacteria | phnP | Phosphonate and phosphinate metab. | Metab. Of other amino acids |
| 1783 | Rhodothermia | nicotinamide phosphoribosyltransferase | Nicotinate and nicotinamide metab. | Metab. Of cofactors and vitamins |
| 1783 | Rhodothermia | PhoH-like protein | Signaling proteins |  |
| 1826 | Actinomycetia | ycsE, yitU, ywtE | Riboflavin metab. | Metab. Of cofactors and vitamins |
| 1834 | Bacilli | phnP | Phosphonate and phosphinate metab. | Metab. Of other amino acids |
| 1836 | Bacteroidia | GH136 lacto-N-biosidase | Glycosyl hydrolases | Carbohydrate metab. |
| 1854 | Gammaproteobacteria | DHFR, folA; dihydrofolate reductase | Folate biosynthesis | metab. Of cofactors and vitamins |
| 1860 | Alphaproteobacteria | Glycosyl hydrolases family 16 | Glycosyl hydrolases | Carbohydrate metab. |
| 1869 | Bacteroidia | GH136 lacto-N-biosidase | Glycosyl hydrolases | Carbohydrate metab. |
| 1872 | Chloroflexia | glf; UDP-galactopyranose mutase | Galactose metab. | Carbohydrate metab. |
| 1894 | Rhodothermia | Concanavalin A-like lectin/glucanases superfamily | - | - |
| 1894 | Rhodothermia | Mannosyl-glycoprotein endo-beta-N-acetylglucosaminidase | Other glycan degradation | Glycan biosynthesis and metab. |
| 1894 | Rhodothermia | DHFR, folA; dihydrofolate reductase | Folate biosynthesis | metab. Of cofactors and vitamins |
| 1894 | Rhodothermia | NTE family protein | - | - |
| 1894 | Rhodothermia | nicotinamide phosphoribosyltransferase | Nicotinate and nicotinamide metab. | Metab. Of cofactors and vitamins |
| 1894 | Rhodothermia | phnP; phosphoribosyl 1,2-cyclic phosphate phosphodiesterase | Phosphonate and phosphinate metab. | Metab. Of other amino acids |
| 1913 | Rhodothermia | HTH-type transcriptional regulator | Nicotinate and nicotinamide metab. | Metab. Of cofactors and vitamins |
| 1913 | Rhodothermia | PhoH-like protein | Signaling proteins | 0 |
| 1916 | Gammaproteobacteria | pseH | Amino sugar and nucleotide sugar metab. | Carbohydrate metab. |
| 1932 | Rhodothermia | phnP | Phosphonate and phosphinate metab. | Metab. Of other amino acids |
| 1935 | Bacteroidia | GH136 lacto-N-biosidase | Glycosyl hydrolases | Carbohydrate metab. |
| 1936 | Gammaproteobacteria | nicotinamide phosphoribosyltransferase | Nicotinate and nicotinamide metab. | Metab. Of cofactors and vitamins |
| 1936 | Gammaproteobacteria | GH135 alpha-1,4-galactosaminogalactan hydrolase | Glycosyl hydrolases | Carbohydrate metab. |
| 1942 | Thermoanaerobaculia | GH8 chitosanase; cellulase; licheninase | Glycosyl hydrolases | Carbohydrate metab. |
| 1953 | Gammaproteobacteria | Lactonase | - | - |
| 1953 | Gammaproteobacteria | Concanavalin A-like lectin/glucanases superfamily | - | - |
