# Supplementary material for: Viral Community Structure and Potential Functions in the Dried-Out Aral Sea Basin Change along a Desiccation Gradient
Source: mSystems. 2023 Jan 10;8(1):e00994-22. doi: 10.1128/msystems.00994-22 (PMC9948696; doi:10.1128/msystems.00994-22)
Supplement: TABLE S4 [file msystems.00994-22-s0007.docx]

**Supplementary Table S4 Details about manually curated of selected AMGs**

| vOTU ID | ORF | Annotation | Conserved domain | Phyre2 | | | |
| --- | --- | --- | --- | --- | --- | --- | --- |
|  |  |  |  | Alignment hit | Conf. (%) | Cov (%) | id (%) |
| 1783 | 1783_42 | nicotinamide phosphoribosyltransferase | PRK09198 | nicotinamide phosphoribosyltransferase | 100 | 79 | 34 |
| 1916 | 1916_6 | pseH | UDP-4-amino-4 | dp-4-amino-4,6-dideoxy-n-acetyl-beta-l-altrosamine n- | 100 | 91 | 27 |
| 1754 | 1754_92 | Aldo/keto reductase family | Aldo-keto reductase (AKR) | oxidoreductase | 100 | 88 | 50 |
| 1754 | 1754_97 | quinolinate synthase | Quinolinate synthetase A | quinolinate synthetase a | 100 | 72 | 33 |
| 1936 | 1936_227 | GH135 alpha-1,4-galactosaminogalactan hydrolase | Spherulation-specific | crystal structure of aspergillus clavatus sph3 | 100 | 65 | 27 |
| 1894 | 1894_42 | Mannosyl-glycoprotein endo-beta-N-acetylglucosaminidase | Mannosyl-glycoprotein | n-acetylglucosaminidase | 100 | 45 | 29 |
| 1894 | 1894_66 | DHFR, folA; dihydrofolate reductase | DHFR | dihydrofolate reductase | 100 | 94 | 41 |
| 194 | 194_12 | nicotinamide phosphoribosyltransferase | PRK09198 | nicotinamide phosphoribosyltransferase | 100 | 79 | 33 |
| 194 | 194_222 | CBH2, cbhA; cellulose 1,4-beta-cellobiosidase | FN3 | receptor-type tyrosine-protein phosphatase f | 99 | 53 | 18 |
| 194 | 194_233 | DHFR, folA; dihydrofolate reductase | DHFR | dihydrofolate reductase | 100 | 96 | 39 |
| 1709 | 1709_7 | horismite mutase | Chorismate mutase type II | horismate mutase | 100 | 96 | 39 |
| 311 | 311_16 | branched-chain amino acid transport system permease | TM_PBP1_branched-chain-AA | ABC transporter involved in vitamin B12 uptake, BtuC | 97 | 84 | 18 |
| 311 | 311_17 | branched-chain amino acid transport system permease | TM_PBP1_branched-chain-AA | hemin transport system permease protein hmuu | 97 | 87 | 17 |
| 311 | 311_23 | N-formylglutamate amidohydrolase | N-formylglutamate amidohydrolase | Phosphorylase/hydrolase-like | 100 | 95 | 35 |
| 311 | 311_24 | glutamine synthetase | Glutamine synthetase | glutamine synthetase | 100 | 94 | 40 |
| 419 | 419_10 | asnB, ASNS; asparagine synthase | Asparagine synthase | asparagine synthetase b | 100 | 90 | 30 |
| 860 | 860_30 | 7-cyano-7-deazaguanine reductase | queF(Superfamily) | adph-dependent 7-cyano-7-deazaguanine reductase | 100 | 91 | 52 |
| 828 | 828_14 | alkaline phosphatase D | Phosphodiesterase/alkaline | alkaline phosphatase d | 100 | 50 | 18 |
| 812 | 812_23 | enolase | enolase | enolase | 100 | 100 | 73 |
| 761 | 761_43 | Sulfotransferase family | Sulfotransferase | PAPS sulfotransferase | 98 | 82 | 18 |
| 1112 | 1112_17 | Cellulase GH5 | Glycosyl hydrolases | endoglucanase | 100 | 46 | 50 |
| 1112 | 1112_88 | Cellulase GH5 | Glycosyl hydrolases | endoglucanase | 100 | 44 | 45 |
| 1156 | 1156_3 | Cellulase GH5 | Glycosyl hydrolases | endoglucanase | 100 | 54 | 48 |
| 1234 | 1234_10 | GH5 endo-beta-1,4-glucanase / cellulase | Glycosyl hydrolases | endoglucanase | 100 | 40 | 37 |
| 1388 | 1388_13 | GH87 mycodextranase | Pectate lyase | preneck appendage protein | 100 | 78 | 16 |
| 1388 | 1388_14 | CE4 acetyl xylan esterase | Catalytic NodB homology | poly-beta-1,6-n-acetyl-d-glucosamine n-deacetylase | 100 | 54 | 25 |
| 1511 | 1511_90 | GH3 beta-glucosidase | beta-glucosidase BglX | thermostable beta-glucosidase b | 100 | 95 | 62 |
| 1353 | 1353_32 | GH6 endoglucanase | Glycosyl hydrolases | Glycosyl hydrolases family 6, cellulases | 100 | 52 | 43 |
| 1495 | 1495_6 | GH26 beta-mannanase | Glycosyl hydrolase | beta-glycanases | 100 | 13 | 39 |
| 1684 | 1684_24 | alkaline phosphatase D | metallophosphatase | alkaline phosphatase d | 100 | 64 | 22 |
| 1942 | 1942_60 | GH8 chitosanase | Glycosyl hydrolases | Glucanase | 100 | 36 | 40 |
