# Supplementary material for: Viral Community Structure and Potential Functions in the Dried-Out Aral Sea Basin Change along a Desiccation Gradient
Source: mSystems. 2023 Jan 10;8(1):e00994-22. doi: 10.1128/msystems.00994-22 (PMC9948696; doi:10.1128/msystems.00994-22)
Supplement: TABLE S5 [file msystems.00994-22-s0008.docx]

**Supplementary Table S5. Number of high-quality reads, assembled contigs and detected viral contigs**

| Sample ID | Number of hiqh quality PE reads | Number of assembled contigs (>10 kB) | Detected viral contigs* |
| --- | --- | --- | --- |
| AS1 | 55,354,273 | 3071 | 227 |
| AS2 | 55,325,180 | 4305 | 243 |
| AS3 | 60,701,071 | 3652 | 373 |
| AS4 | 61,226,670 | 5311 | 213 |
| AS5 | 56,821,217 | 3896 | 232 |
| AS6 | 49,811,404 | 2791 | 242 |
| AS7 | 40,999,012 | 2417 | 131 |
| AS8 | 46,834,846 | 2784 | 214 |
| AS9 | 39,583,252 | 1916 | 280 |

*Viral contigs were identified by using VirSorter v2.2.3 and nontargeted virus sequence discovery pipeline as described by Paez-espino et al., 2017
